# Silicon‐Based Azo Compound‐Mediated CO Activation and N_2_ Release

**DOI:** 10.1002/anie.202517538

**Published:** 2025-09-26

**Authors:** Da Jin, Alexander Hinz, Xiaofei Sun, Peter W. Roesky

**Affiliations:** ^1^ Institute of Inorganic Chemistry Karlsruhe Institute of Technology (KIT) Kaiserstr. 12 76131 Karlsruhe Germany; ^2^ Institute of Nanotechnology (INT) Karlsruhe Institute of Technology (KIT) Kaiserstr. 12 76131 Karlsruhe Germany

**Keywords:** Azo compounds, CO activation, Main group, Silicon–nitrogen multiple bonding, Silylene

## Abstract

Silicon‐substituted azo compounds featuring Si─N═N─Si linkages remain elusive in main‐group chemistry. Herein, we report the synthesis of a silicon‐based azo compound generated directly from a mixed‐valent silaiminyl–silylene precursor and a bulky organic azide. Unlike classical iminosilane formation, this reaction affords a thermally stable Si(IV)–azo species. Upon treatment with carbon monoxide (CO), this compound undergoes N_2_ extrusion, complete C≡O bond cleavage, and formation of a formal Si(II)/Si(IV) product in which an oxygen atom bridges both silicon centers. Notably, the transformation incorporates the carbon atom into a DippNC byproduct via ligand rearrangement. A similar transformation occurs upon reaction with Fe(CO)_5_, wherein N_2_ release and CO cleavage also occur, but with the resulting Fe(CO)_4_ fragment coordinating to the silicon center. These results demonstrate a rare example of silicon based azo‐mediated small‐molecule activation and highlight the potential of silicon‐based systems for multi‐electron redox chemistry typically associated with transition metals.

## Graphical abstract

A rare silicon(IV)‐based azo compound featuring a Si─N═N─Si linkage was synthesized via the direct reaction of an organoazide with a silaiminyl–silylene precursor. This compound undergoes an unusual CO activation process, resulting in N_2_ release and complete cleavage of the C≡O bond, forming a Si(II)/Si(IV) product bridged by an oxygen atom and an organic isonitrile.

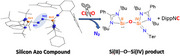

## Introduction

**Figure 1:**
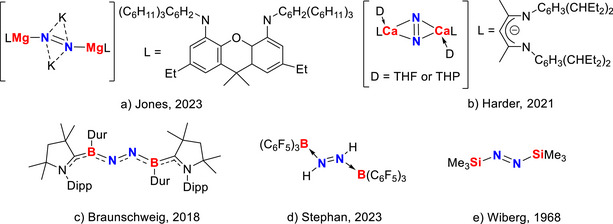
Selected examples of main‐group N_2_‐derived species.

Azo compounds (R─N═N─R′), characterized by a nitrogen–nitrogen double bond, are well‐established functional groups in organic synthesis,^[^ ^1^, ^2^, ^3^ ^]^ materials chemistry,^[^ ^4^, ^5^, ^6^ ^]^ and redox‐responsive systems.^[^ ^7^, ^8^, ^9^ ^]^ A related structural motif can be established by metal‐mediated activation of dinitrogen, enabling the formation of transition‐metal^[^ ^10^, ^11^, ^12^, ^13^ ^]^ and f‐block complexes^[^ ^14^, ^15^, ^16^ ^]^ featuring metal‐bridged azo units (M─N═N─M). More recently, main‐group elements have been explored as platforms for stabilizing azo functionalities. However, such systems remain scarce, primarily due to intrinsic limitations such as reduced redox flexibility and the absence of π‐backbonding. Despite these challenges, a limited number of structurally characterized examples have been reported, including species derived from N_2_ activation by Mg,^[^ ^17^ ^]^ Ca,^[^ ^18^, ^19^ ^]^ Sr,^[^ ^20^ ^]^ and B,^[^ ^21^, ^22^, ^23^ ^]^ as well as from alternative precursors such as borane stabilized^[^ ^24^ ^]^ and phosphine coordinated diazenes (Figure 1a–d).^[^ ^25^, ^26^ ^]^

Within the realm of silicon chemistry, azo derivatives are still underdeveloped. A notable example bis(trimethylsilyl)diazene (Figure 1e) was reported by Wiberg in 1968, forming from the low‐temperature reaction of *p*‐tosylazide with lithium tris(trimethylsilyl)hydrazide.^[^ ^27^ ^]^ This azo compound readily dimerizes (*T*_decomp_ < –40 °C) with concomitant N_2_ extrusion to yield tetrakis(trimethylsilyl)hydrazine. Its trans configuration was later confirmed by Veith and Bärnighausen through single‐crystal X‐ray diffraction.^[^ ^28^ ^]^ Due to the low thermal stability, its reactivity remained unexamined until 2014, when Schulz and co‐workers revised its structure and reported the related trisilylated diazenium ion [(Me_3_Si)_2_N═N─SiMe_3_]⁺.^[^ ^29^ ^]^ Since then, no new examples of structurally defined silicon azo compounds have been reported.

Herein, we report a thermally stable silicon(IV) azo compound synthesized directly from a mixed‐valent silaiminyl–silylene precursor and a bulky organoazide, forming a Si─N═N─Si linkage. Remarkably, the resulting Si(IV) azo compound can react cleanly with CO under mild conditions—a rare transformation for a Si(IV) species. This process involves complete cleavage of the CO triple bond with concurrent N_2_ extrusion and an organic isonitrile formation. These findings demonstrate that main‐group species—when appropriately designed—can mediate multi‐electron, multi‐bond activation events traditionally reserved for transition metals.

## Results and Discussion

**Scheme 1:**
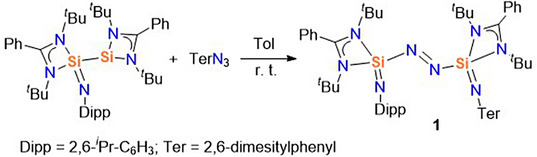
Synthesis of the bis(silaiminyl)azo compound **1**.

The silaiminyl–silylene compound [LSi─Si(NDipp)L] (L = PhC(N*^t^* Bu)_2_, Dipp = 2,6‐diisopropylphenyl) was synthesized according to a method previously reported by our group.^[^ ^30^ ^]^ Treatment of this compound with one equivalent of [TerN_3_] (Ter = 2,6‐bis(2,4,6‐trimethylphenyl)phenyl)^[^ ^31^ ^]^ in toluene at room temperature led to an immediate color change of the solution from yellow to dark red. This reaction afforded the bis(silaiminyl)azo compound **1** (Scheme 1) as red crystals in 66% yield. During the transformation, the terminal nitrogen atoms (═N═N) of the azide group are inserted into the Si─Si bond, forming a bridging azo unit. Simultaneously, the remaining [Ter–N] fragment is incorporated as a [TerN═Si] silaiminyl moiety. This reactivity contrasts with the silylene‐azide reactions yielding more common iminosilanes (R_2_Si═NR)^[^ ^32^, ^33^, ^34^, ^35^ ^]^ or unusual nitrenes^[^ ^36^ ^]^ via N_2_ elimination.

In the ^1^H NMR spectrum of **1**, four singlet signals were observed at *δ* 2.62, 2.22, 1.04, and 0.68 ppm. The first two are attributed to the ortho‐ and para‐methyl groups of the mesityl substituents, while the latter two correspond to the tert‐butyl groups. A septet at *δ* 4.41 ppm and a doublet at *δ* 1.52 ppm were observed for the C*H* and methyl protons of the *^i^* Pr group on the Dipp substituent. The ^29^Si{^1^H} NMR spectrum displays two resonances at *δ* −102.8 and −109.5 ppm with nearly equal intensities, indicating that the two silicon atoms reside in comparable chemical environments. Both signals appear significantly upfield relative to those of the starting material (*δ* −61.7 and 31.8 ppm),^[^ ^30^ ^]^ consistent with increased shielding at the silicon centers due to coordination by electron‐rich nitrogen donors. The observed chemical shifts are consistent with values reported for tetracoordinate Si(IV) species bearing multiple nitrogen ligands.^[^ ^33^, ^37^, ^38^ ^]^

**Figure 2:**
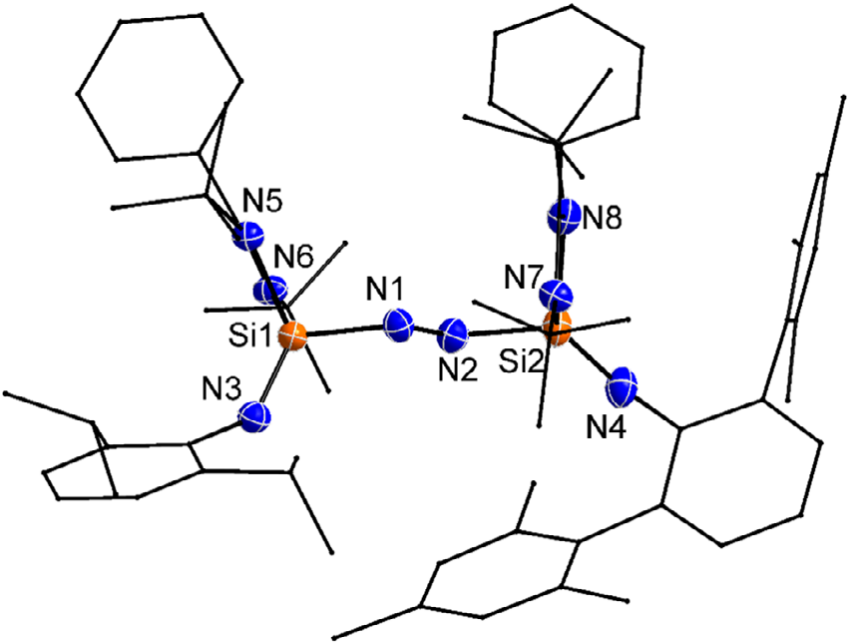
Molecular structure of **1** in the solid state. All hydrogen atoms are omitted for clarity. Selected bond lengths (Å) and bond angles [°]: N1─N2 1.236(7), Si1─N1 1.797(4), Si1─N3 1.573(2), Si1─N5 1.815(2), Si1─N6 1.833(2), Si2─N2 1.813(4), Si2─N4 1.553(2), Si2─N7 1.813(2), Si2─N8 1.817(2); N1─Si1─N3 111.38(15), N2─N1─Si1 119.2(4), N1─N2─Si2 115.2(3), N2─Si2─N4 117.05(14).

The molecular structure of compound **1** was confirmed by single‐crystal X‐ray diffraction analysis (Figure 2). The central azo unit adopts a trans configuration, with the two silicon atoms (Si1 and Si2) positioned on opposite sides of the N═N bridge. Each silicon center is coordinated to one azo nitrogen and three additional nitrogen donors from the supporting ligands, resulting in a distorted tetrahedral geometry. The N1═N2 bond length is 1.236(7) Å, consistent with double‐bond distances found in reported azo compounds.^[^ ^13^, ^21^, ^39^ ^]^ Notably, the Si1─N3 (1.573(2) Å) and Si2─N4 (1.553(2) Å) bond lengths are significantly shorter than the other Si─N distances in the structure (1.797–1.833 Å), suggesting double‐bond character, and are comparable to those reported of silaiminyl (Si═NR) compounds.^[^ ^33^, ^38^ ^]^ The substantial steric protection provided by the Dipp and Ter substituents on N3 and N4 likely contributes to the kinetic stabilization of this rare Si─N═N─Si framework.

Given the azo functionality of compound **1**, the photostability was investigated to assess potential cis‐trans isomerization. However, no conformational changes were observed under irradiation. Thermal stability studies of compound **1** revealed that decomposition commences at approximately 180 °C, as monitored by ^1^H NMR spectroscopy in C_6_D_6_ using a J. Young NMR tube. Despite efforts to crystallize the decomposition products for further structural characterization, no crystalline material was obtained. Furthermore, side‐on bridging dinitrogen complexes of transition metals have previously been shown to react with CO in multi‐insertion pathways, producing oxamidide species (─N═C(O)─C(O)═N─) via formal double CO insertion into the N═N unit,^[^ ^40^, ^41^ ^]^ or forming carbonyl adducts accompanied by N_2_ release.^[^ ^42^ ^]^ Inspired by these precedents, we explored whether the central azo moiety in compound **1** could undergo a similar transformation upon exposure to CO.

**Scheme 2:**
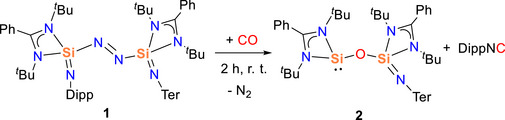
The reaction of compound **1** with CO.

When a toluene solution of compound **1** was exposed to CO at ambient pressure, a gradual color change from dark red to yellow was observed (Scheme 2). The ^29^Si{^1^H} NMR spectrum revealed two distinct resonances at *δ* −22.1 and −105.7 ppm, indicating a significant change in the electronic environment at one of the silicon centers. Attempts to isolate single crystals suitable for X‐ray diffraction were unsuccessful. Nevertheless, clean NMR spectra were obtained for the reaction mixture. ^1^H and ^13^C{^1^H} NMR analyses confirmed the formation of DippNC as a byproduct (Figures S4 and S5).^[^ ^43^, ^44^ ^]^ The high boiling point and viscosity of DippNC likely hindered crystallization.

Although single‐crystal data could not be obtained, the structure of compound **2** was inferred from a combination of experimental NMR data and DFT‐calculated ^29^Si{^1^H} NMR chemical shifts (*δ* −11 and −101 ppm, Gaussian16, PBE0/def2‐SVP/D3^[^ ^45^ ^]^). Compound **2** is an siloxy‐silylene, and the resonance at *δ* −22.1 ppm is comparable to those reported values for monosilylene alkoxide [LSiO*^i^* Pr] (*δ* −13.5 ppm)^[^ ^46^ ^]^ and disilylenoxane [LSi─O─SiL] (*δ* −16.1 ppm) bearing the same amidinato substituent.^[^ ^47^ ^]^

The observed CO activation here is distinct from previously reported reactions involving other bis‐silicon species, such as the 1,2‐disilylene,^[^ ^48^ ^]^ bis(silylenes)^[^ ^49^ ^]^ and disilienes.^[^ ^50^, ^51^, ^52^ ^]^ However, it resembles reactivity of N_2_ complexes with poor back‐donation.^[^ ^17^, ^18^, ^19^, ^20^ ^]^ The formation of **2** from **1** was also studied computationally (see section 5 in the Supporting Information, Figures S35–S39) with model compounds, that comprise methyl instead of *tert*‐butyl as well as phenyl for Dipp and terphenyl groups. This allowed to corroborate a plausible but complex reaction pathway that proceeds stepwise via addition of CO, elimination of N_2_ and elimination of the isonitrile. However, despite all efforts, some transition states eluded identification. The addition of CO can proceed as a [2 + 2] cycloaddition with the Si═N(Dipp) bond yielding a SiNCO cycle (Figure S36, Int2). Subsequent migration of the iminosilyl group along the N═N moiety then facilitates elimination of N_2_ and affording a Si─Si bonded intermediate (Figure S37, Int5). For the isonitrile elimination, two paths may be viable. Either, an attack of the iminosilyl group to the O atom breaks the SiNCO cycle, extrudes the O atom and leads to a SiNC cycle. This then rearranges to a linear SiCNR moiety, from which the isonitrile CNR can dissociate. Another plausible path from Int5 is via isomerisation to silyl‐silylene Int9 with a bridging amide moiety, from which the isonitrile can be eliminated in a cycloreversion reaction that leaves a silanone moiety (Si═O), which is then internally rearranged to a Si_2_NO cycle which opens to yield the oxide‐bridged iminosilyl‐silylene product.

**Scheme 3:**
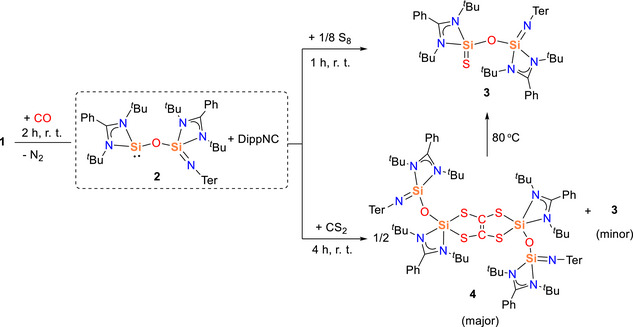
The trapping reactions of **2** by S_8_ and CS_2_.

To validate the formation of silylene compound **2**, additional trapping experiments were performed. Due to the presence of nucleophilic DippNC, only a limited range of electrophilic trapping agents could be used. Elemental sulfur (S_8_) was selected first for its compatibility (Scheme 3). Upon addition of S_8_ to a C_6_D_6_ solution containing compound **2** and DippNC, the ^1^H NMR spectrum showed subtle shifts for four singlets appearing at *δ* 2.60, 2.30, 1.25, and 0.98 ppm, compared to *δ* 2.63, 2.32, 1.19, and 0.88 ppm in compound **2**. Signals corresponding to DippNC remained detectable in the mixture. The ^29^Si{^1^H} NMR spectrum displayed two new resonances at *δ* −36 and −108 ppm. The resonance at *δ* −36 ppm is shifted upfield relative to the corresponding signal in compound **2** (*δ* −22.1 ppm), indicating increased shielding consistent with sulfur coordination and formation of a Si═S moiety. This value is comparable to that reported for a bis(silanethione) compound (*δ* −37.29 ppm).^[^ ^53^ ^]^

**Figure 3:**
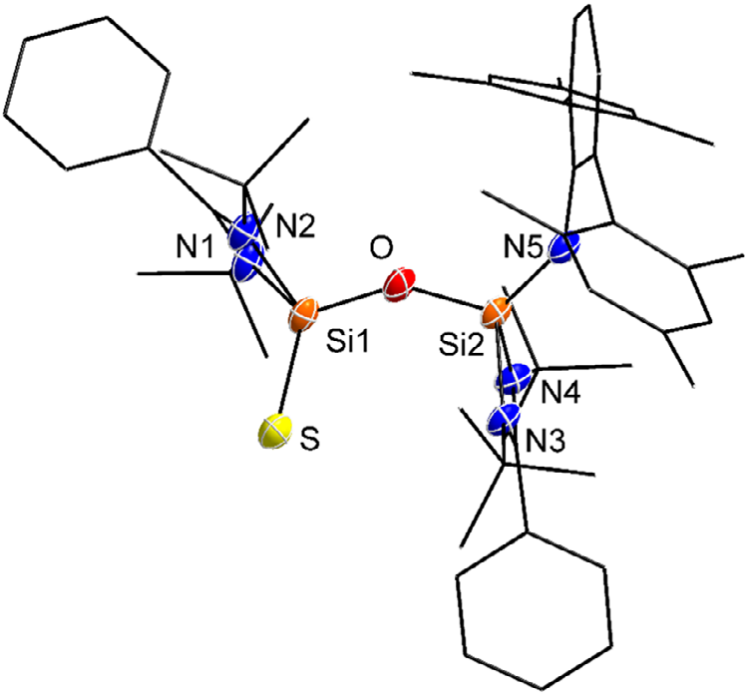
Molecular structure of **3** in the solid state. All hydrogen atoms are omitted for clarity. Selected bond lengths (Å) and bond angles [°]: S─Si1 1.9763(13), Si1─O 1.621(2), Si2─O 1.636(2); O─Si1─S 120.74(9), Si1─O─Si2 146.4(2), N1─Si1─N2 71.86(12), N3─Si2─N4 71.79(12).

The addition of elemental sulfur, interestingly, improved the crystallinity of the reaction product, despite the continued presence of DippNC. Single crystals suitable for X‐ray diffraction were obtained by layering pentane over a diethyl ether solution of the reaction mixture. The molecular structure of compound **3** (Figure 3) reveals a heteroleptic disilicon core bridged by an oxygen atom and capped by a terminal sulfur, forming a Si─O─Si═S framework. The two amidinate ligands are positioned trans to each other across the Si─O─Si core. Si1 is tetra‐coordinated by two nitrogen atoms (N1 and N2) from the amidinate ligand, the bridging oxygen, and a terminal sulfur atom. The Si1═S bond length of 1.9763(13) Å supports a double‐bond character (1.95–2.08 Å)^[^ ^54^, ^55^ ^]^ and closely matches those in the previously reported base‐stabilized silanethiones, for example [LSi(═S)Si(SiMe_3_)_3_] (1.9996(6) Å),^[^ ^56^ ^]^ [LSi(═S)N(SiMe_3_)_2_] (1.987(8) Å),^[^ ^57^ ^]^ [LSi(═S)S*^t^* Bu] (1.984(8) Å),^[^ ^58^ ^]^ the resorcinolate SiCSi pincer bridged bis(silanethione) (1.968(2) and 1.978(2) Å)^[^ ^53^ ^]^ and the silanoic thioeaster (1.980(2) Å).^[^ ^59^ ^]^ The Si─O bond distances (Si1─O 1.621(2) Å; Si2─O 1.636(2) Å) are typical for Si─O single bonds and are comparable to those found in siloxysilylene compounds (1.631–1.655 Å).^[^ ^47^, ^60^ ^]^ Si2 is also tetra‐coordinated, bonded to three nitrogen donors (N3, N4, N5) and the bridging oxygen. The Si1─O─Si2 angle in compound **3** is 146.4(2)°, falling between the values reported for disilylenoxanes [LSi─O─SiL] (159.88(15)°)^[^ ^47^ ^]^ and [L'Si(H)─O─Si(L'H)] (137.08(5)°).^[^ ^60^ ^]^

To further confirm the formation of silylene **2**, carbon disulfide (CS_2_) was employed as a probe reagent (Scheme 3). Upon addition of CS_2_ to a toluene solution containing silylene **2** and DippNC, a rapid color change from yellow to red was observed. Crystallization from this mixture in diethyl ether afforded compound **4**—a dimeric product featuring a central C═C double bond flanked by four sulfur atoms, forming a fully substituted tetrathioethene core. Each sulfur atom bridges to a silicon center bearing a bulky nitrogen‐donor ligand, resulting in a Si─O─Si─[S─C─S]_2_─Si─O─Si framework. This transformation contrasts with most previously reported reactions of silylenes with CS_2_, which typically proceed via carbon coordination to the silylene to form thiocarbonyl‐stabilized adducts (R_2_Si←C═S), followed by the generation of various sila–C,S–containing heterocycles.^[^ ^61^, ^62^, ^63^, ^64^ ^]^ Notably, none of these pathways involve C═C bond formation leading to the ethenetetrathiolate anion form, [C_2_S_4_]^4−^. Among *p*‐block elements, such transformations have so far been limited to several examples, such as heavier group 14 species, stannylenes,^[^ ^65^, ^66^ ^]^ and more recently, aluminum^[^ ^67^ ^]^ and gallium complexes.^[^ ^68^ ^]^

**Figure 4:**
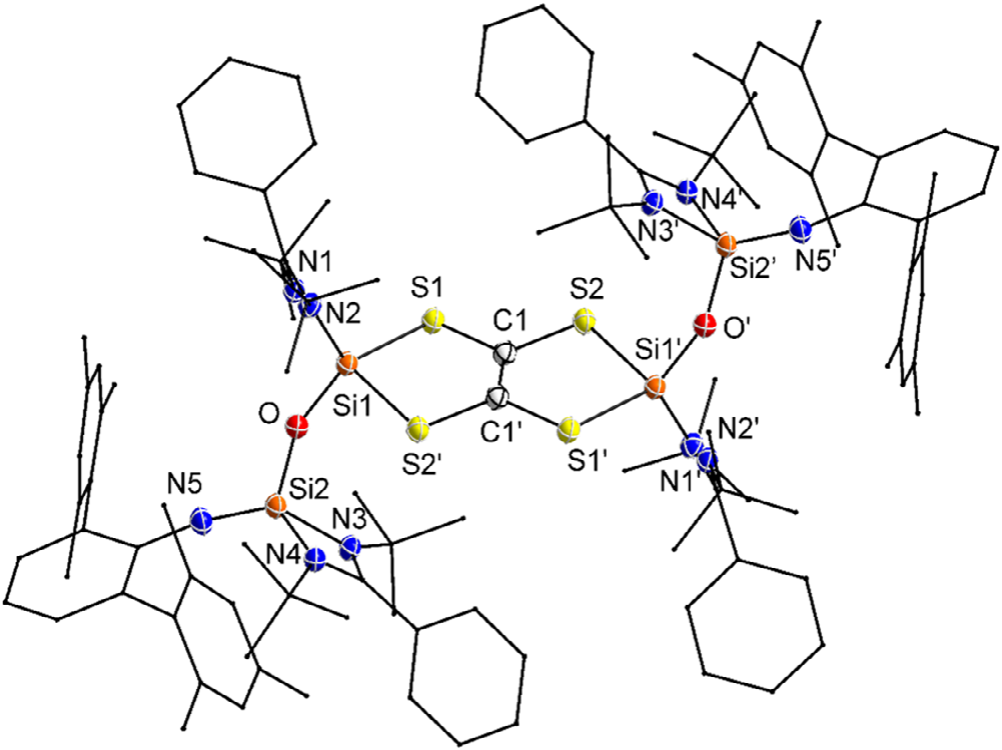
Molecular structure of **4** in the solid state. All hydrogen atoms are omitted for clarity. Selected bond lengths (Å) and bond angles [°]: S1─Si1 2.1867(9), Si1─S2’ 2.2588(11), Si1─O 1.638(2), S1─C1 1.759(3), S2─C1 1.752(2), Si2─O 1.636(2), C1─C1’ 1.332(5); C1─S1─Si1 104.04(9), S1─Si1─S2’ 90.70(4), C1’─S2’─Si1 101.50(10), C1─C1’─S2’ 122.4(3), C1’─C1─S1 120.4(3), S1─C1─S2 117.10(15), Si1─O1─Si2 154.67(13).

Single‐crystal X‐ray diffraction of compound **4** confirmed a dimeric structure composed of two fused five‐membered Si─S─C═C─S─ rings (Figure 4). The sum of the inner angels of the five‐membered ring is 538.64°, indicating a nearly planar geometry. Within the supporting ligand framework, the Si─O─Si angle increases to 154.67°, suggesting some ring strain release. The C═C bond length (1.332(5) Å) is comparable to that reported for tin analogues featuring the same core (1.342(9) Å and 1.346(6) Å).^[^ ^65^, ^66^ ^]^ The Si─S bond lengths (Si1–S1 2.187 Å and Si1–S2' 2.259 Å) are significantly longer than the Si═S double bond in compound **3** (1.9763(13) Å), and are comparable to reported Si─S single bonds (2.12–2.33 Å).^[^ ^61^, ^62^, ^63^ ^]^

NMR analysis of the isolated crystals of compound **4** consistently showed minor signals assignable to compound **3** (Figures S13 and S14). Further studies revealed that **4** gradually decomposes to **3** at room temperature and completely converts upon heating, preventing acquisition of a clean NMR spectrum for pure **4** (Figure S16). Throughout this process, the solution color shifted gradually from yellow to red. Notably, the NMR spectra exhibited no additional signals beyond those corresponding to **3** and unreacted **4**. We attribute the observed color change to the formation of a volatile, colored sulfur‐containing byproduct, which dissolves and imparts color to the solution. The lack of detectable NMR signals is consistent with both its high volatility and low concentration in solution.

**Scheme 4:**
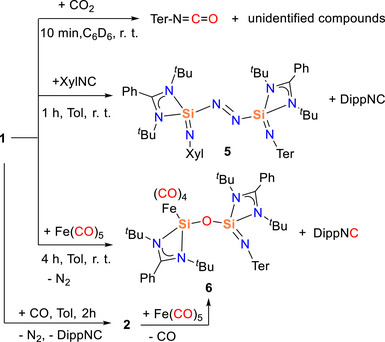
The reactivity of compound **1** with CO_2_, XylNC, and Fe(CO)_5_.

Motivated by the unique reactivity of compound **1** with CO, we next examined its behavior toward CO_2_ and XylNC (Xyl = 2,6‐dimethylphenyl), which can be considered as isoelectronic analogues of CO, to assess the scope and selectivity of the transformation (Scheme 4). An NMR‐scale reaction of **1** with CO_2_ in C_6_D_6_ showed an immediate color change from red to colorless. Analysis of the reaction mixture revealed the formation of TerNCO, confirmed by both NMR spectroscopy (Figure S17)^[^ ^69^, ^70^ ^]^ and single‐crystal X‐ray diffraction (Figure S34). In addition to TerNCO, other unidentified species were observed in the crude mixture, but attempts to isolate them were unsuccessful.

Similarly, the reaction of compound **1** with XylNC was carried out at room temperature in C_6_D_6_. The ^1^H NMR spectrum indicated the formation of DippNC, while the ^2^⁹Si{^1^H} NMR spectrum showed only minor shifts (*δ* −99.1 and −108.3 ppm), consistent with that of compound **1**. Upon concentration of the reaction mixture, orange crystals of compound **5** were obtained and structurally characterized by X‐ray diffraction (Figure S32). The structure revealed substitution of the Dipp‐N group by the Xyl‐N group, without further transformation—even upon heating to 80 °C. In contrast, reactions with *^t^* BuNC and DippNC showed no observable reactivity under identical conditions.

To further investigate the CO‐related reactivity of compound **1**, iron pentacarbonyl (Fe(CO)_5_) was employed as a model transition‐metal carbonyl reagent.^[^ ^71^ ^]^ Treatment of compound **1** with one equivalent of Fe(CO)_5_ in toluene at room temperature resulted in a gradual color change from red to yellow. Concentration of the solution yielded compound **6** as yellow crystals—a novel Fe(CO)_4_‐ligated silicon–oxygen‐bridged complex. This transformation parallels the previously observed reaction of **1** with free CO. In both cases, bond reorganization occurs via N_2_ extrusion and formation of DippNC, while the Fe(CO)_5_ acts as an in situ CO donor. The resulting Fe(CO)_4_ fragment then coordinates to the electron‐rich silicon site. Notably, when compound **1** was pre‐reacted with CO to form silylene intermediate **2**, subsequent addition of Fe(CO)_5_ also led to compound **6**, indicating a convergent reaction pathway and supporting the role of **2** as a key intermediate (Scheme 4).

In the ^2^⁹Si{^1^H} NMR spectrum of compound **6**, two distinct signals were observed at *δ* 35.8 ppm (Si─Fe) and *δ* –113.1 ppm (Si═N). The former, assigned to the Fe‐bound Si atom, is in good agreement with the value reported for the related [{LSiO*^t^* Bu}Fe(CO)_4_] complex (*δ* 40.3 ppm).^[^ ^72^ ^]^ In the ^13^C{^1^H} NMR spectrum, the *C*O ligands coordinated to the Fe center resonate at *δ* 217.6 ppm, comparable to those found in other silylene–Fe(CO)_4_ complexes.^[^ ^72^, ^73^, ^74^ ^]^ The IR spectrum exhibits four $\tilde{\nu }$(CO) stretching bands at 2026, 1944, 1914, and 1898 cm^−1^, indicating a low‐symmetry environment around the Fe(CO)_4_ unit. This deviates from the more typical three‐band pattern observed in higher symmetrical silylene–Fe(CO)_4_ complexes.^[^ ^72^, ^73^, ^74^ ^]^

**Figure 5:**
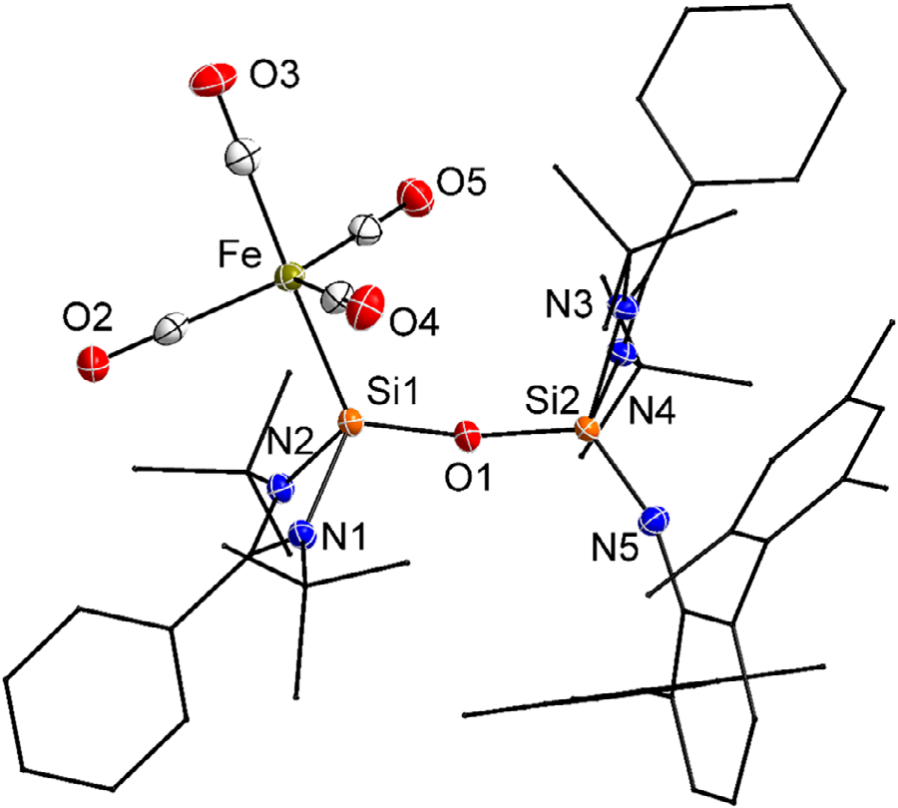
Molecular structure of **6** in the solid state. All hydrogen atoms are omitted for clarity. Selected bond lengths (Å) and bond angles [°]: Fe─Si1 2.2472(6), Si1─O1 1.6209(13), Si1─N1 1.830(2), Si1─N2 1.8376(15), Si2─O1 1.6308(13), Si2─N3 1.8342(15), Si2─N4 1.822(2), Si2─N5 1.571(2); Si1─O1─Si2 166.35(9), O1─Si1─Fe 121.59(5), N1─Si1─N2 71.52(7), N3─Si2─N4 71.83(7).

Single‐crystal X‐ray diffraction analysis of compound **6** (Figure 5) reveals a bent Si─O─Si bridge connecting the two silicon atoms, with a bond angle of 166.35°, larger than those found in compounds **3** (146.4°) and **4** (154.7°). The Si─Fe bond length of 2.247 Å closely matches those reported for related complexes such as [(O*^t^* Bu)LSiFe(CO)_4_] (2.237(7) Å)^[^ ^72^ ^]^ and [{Me_2_NC(N*^t^* Bu)(NDipp)}(H)SiFe(CO)_4_] (2.234(1) Å).^[^ ^75^ ^]^

## Conclusion

In summary, a rare Si(IV)‐based azo compound **1**, featuring the Si(IV)─N═N─Si(IV) linkage, was synthesized via the oxidative addition of an organoazide to a mixed‐valence silaiminyl–silylene precursor. This isolable silicon(IV)‐azo species exhibits special reactivity toward CO, resulting in N_2_ release and complete cleavage of the C≡O bond to form compound **2**—a siloxane‐type structure containing an Si(IV)─O─Si(II) linkage with silicon atoms in distinct oxidation states, along with the formation of DippNC. Although compound **2** could not be crystallized, its formation was supported by subsequent reactivity studies with S_8_ and CS_2_, as well as DFT calculations. The reactivity of isoelectronic analogues of CO, isocyanides, was also investigated; however, only ligand exchange was observed with XylNC. Further reaction of compound **1** with Fe(CO)_5_ demonstrated similar behavior to that with CO. These findings highlight the transition‐metal‐like multi‐bond activation capability of silicon centers in main‐group chemistry.

## Supporting Information

The authors have cited additional references within the Supporting Information.^[^ ^76^, ^77^, ^78^, ^79^ ^]^

## Conflict of Interests

The authors declare no conflict of interest.

## Supporting information



Supporting Information

Supporting Information

Supporting Information

## Data Availability

Deposition Numbers 2477857 (for **1**), 2477858 (for **3**), 2477859 (for **4**), 2477860 (for **5**), 2477861 (for **6**) contain the supplementary crystallographic data for this paper. These data can be obtained free of charge via www.ccdc.cam.ac.uk/data_request/cif., or by emailing data_request@ccdc.cam.ac.uk, or by contacting The Cambridge Crystallographic Data Centre, 12 Union Road, Cambridge CB2 1EZ, UK; fax: +44 1223 336 033. NMR, IR and EA data for this paper are available at radar4chem [https://radar.products.fiz‐karlsruhe.de/] at https://doi.org/10.22000/k6h27ykcjq3ct9rn.
