# Supplementary material for: Silicon‐Based Azo Compound‐Mediated CO Activation and N2 Release
Source: Angew Chem Int Ed Engl. 2025 Sep 26;64(47):e202517538. doi: 10.1002/anie.202517538 (PMC12624318; doi:10.1002/anie.202517538)
Supplement: Supplementary file 1 — Supporting Information [file ANIE-64-e202517538-s001.pdf]

Silicon-Based Azo Compound-Mediated CO Activation and N_2 Release

Da Jin,[a] Alexander Hinz,[a] Xiaofei Sun[a] and Peter W. Roesky*[a][b]

a. Institute of Inorganic Chemistry, Karlsruhe Institute of Technology (KIT), Kaiserstr. 12, 76131 Karlsruhe, Germany
b. Institute of Nanotechnology (INT), Karlsruhe Institute of Technology (KIT), Kaiserstr. 12, 76131 Karlsruhe, Germany
E-mail: roesky@kit.edu

Table of Contents

1. Synthesis and characterization S2

1.1 General procedures S2

1.2 Synthesis of **1** S2

1.3 Synthesis of **2** S3

1.4 Synthesis of **3** S4

1.5 Synthesis of **4** S4

1.6 Synthesis of **5** S5

1.7 Synthesis of **6** S6

2. NMR spectra S7

3. IR spectra S19

4. X-ray crystallographic studies S21

4.1 General methods S21

4.2 Summary of crystal data S22

4.3 Crystal structures S23

5. Computational details S29

6. References S33

1. Synthesis and characterization

1.1 General procedures

All manipulations of water- and air-sensitive compounds were performed with exclusion of moisture and oxygen in flame-dried Schlenk-type glassware either on a dual manifold Schlenk line, interfaced to a high vacuum (10^{-3} mbar) line or in an argon-filled MBraun glove box. All solvents were dried by using a MBraun solvent purification system (SPS 800). C_6D_6 and THF-d_8 were dried over Na-K alloy and degassed by freeze-pump-thaw cycles. NMR spectra were recorded on Bruker spectrometers (Avance Neo 300 MHz, Avance Neo 400 MHz or Avance III 400 MHz) at 298 K. Chemical shifts are referenced internally using signals of the residual protio solvent (^{1}H) or the solvent (^{13}C{^{1}H}) and are reported relative to tetramethylsilane (^{1}H, ^{13}C{^{1}H}). The multiplicity of the signals is indicated as s = singlet, d = doublet, t = triplet, hept = heptet, m = multiplet and br = broad. Assignments were determined on the basis of unambiguous chemical shifts, coupling patterns and ^{13}C DEPT experiments or 2D correlations (^{1}H–^{1}H COSY, ^{1}H–^{13}C HMQC and ^{1}H–^{13}C HMBC). Elemental analyses were carried out with an Elementar Vario Micro cube from Elementar Analysensysteme GmbH. IR spectra were obtained on a Bruker Tensor 37 spectrometer equipped with a room temperature DLaTGS detector, a diamond ATR (attenuated total reflection) unit and a nitrogen-flushed chamber. In terms of their intensity, the signals were classified into different categories (vs = very strong, s = strong, m = medium, w = weak, and sh = shoulder).

The silaiminyl-silylene [LSi–Si(NDipp)L],[1] TerN$_3$,[2] and DippNC[3] were prepared following literature procedures. S_8, CS_2, XylNC, and $Fe(CO)_5$ are commercially available and used without further purification.

1.2 Synthesis of 1

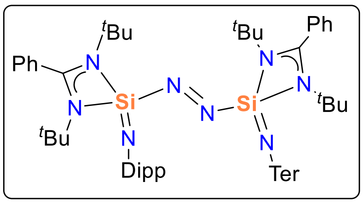

Toluene (20 mL) was added to a mixture of silaiminyl-silylene [LSi–Si(NDipp)L] (0.400 g, 0.576 mmol) and [TerN$_3$] (0.205 g, 0.576 mmol) at room temperature. The reaction mixture rapidly changed color from yellow to dark red. After stirring for 4 h, all volatiles were removed under reduced pressure. The resulting solid residue was redissolved in diethyl ether (4 mL), and the solution was carefully layered with *n*-pentane (15 mL). The resulting biphasic system was stored at −15 °C for two days, affording large, blocky red crystals of the target compound.
Yield (based on crystals): 0.398 g (0.379 mmol), 66%.

Anal. Calcd. for $C_{66}H_{88}N_8Si_2$ (1049.66 g/mol): C, 75.52; H, 8.45; N, 10.68. Found: C, 75.55; H, 8.02; N, 10.34.

^{1}H NMR (400 MHz, C_6D_6, 298 K): δ (ppm) = 7.44 (d, J = 7.6 Hz, 2H, Dipp-*m*-C*H*), 7.37 – 7.32 (m, 1H, Ar), 7.30 – 7.25 (m, 2H, Ar), 7.22 (d, J = 7.3 Hz, 2H, Ar), 7.18 – 7.14 (overlapped with C_6D_6, 1H, Dipp-*p*-H), 7.09 – 7.06 (m, 1H, Ar), 7.04 – 7.01 (m, 1H, Ar), 6.97 – 6.91 (m, 10H, Ar), 4.41 (hept, J = 6.8 Hz, 2H, Dipp-C*H*), 2.62 (s, 12H, Mes-*o*-CH_3), 2.22 (s, 6H, Mes-*p*-CH_3), 1.53 (d, J =

6.8 Hz, 12H, Dipp-CH_3), 1.04 (s, 18H, $C(CH_3)_3$), 0.68 (s, 18H, $C(CH_3)_3$).

$^{13}C\{^1H\}$ NMR (100 MHz, C_6D_6, 298 K): δ (ppm) = 177.6 (N*C*N), 177.5 (N*C*N), 147.5 (C_q), 147.1 (Dipp, C_{ipso}), 143.0 (Mes-*o*-C_q), 140.6 (Dipp-C_q-^{i}Pr), 137.4 (Mes, C_{ipso}), 135.1 (C_q), 134.6 (Mes-*p*-C_q), 130.8 (Ar-*C*H), 130.5 (Ar-*C*H), 130.4 (Ar), 130.3 (C_q), 130.1 (C_q), 129.5 (Ar-*C*H), 128.9 (Ar-*C*H), 128.6 (Ar-*C*H), 128.2 (Ar-*C*H), 127.9 (Mes-*m*-*C*H), 122.8 (Dipp-*m*-*C*H), 117.0 (Dipp-*p*-*C*H), 116.1 (Ar-*C*H), 54.3 ($C(CH_3)_3$), 53.7 ($C(CH_3)_3$), 31.2 ($C(CH_3)_3$), 30.7 ($C(CH_3)_3$), 28.2 ($CH(CH_3)_2$), 24.9 ($CH(CH_3)_2$), 21.7 (Mes-*o*-CH_3), 21.2 (Mes-*p*-CH_3).

$^{29}Si\{^1H\}$ NMR (79.5 MHz, C_6D_6, 298 K): δ (ppm) = –102.8, –109.5.

IR (ATR): $\tilde{\nu}$ (cm^{-1}) = 2958 (s), 2914 (m), 2864 (m), 1494 (vs), 1453 (sh), 1398 (vs), 1366 (s), 1281 (w), 1202 (m), 1096 (m), 1026(w), 928 (w), 850 (w), 790 (m), 746 (m), 707(w), 676 (w), 626 (w), 495 (w).

1.3 Synthesis of 2

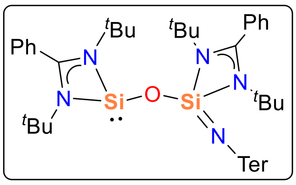

Carbon monoxide (CO) was introduced into a degassed C_6D_6 solution (0.4 mL) of compound **1** (0.030 g, 0.029 mmol) at room temperature. The solution gradually changed color from dark red to light yellow. According to the ^{1}H NMR spectra, the reaction proceeded to completion within 2 h, with concurrent formation of DippNC. Attempts to isolate compound **2** by crystallization were unsuccessful, and it could not be separated from DippNC.

Yield (based on NMR spectra): quantitative.

^1H NMR (400 MHz, C_6D_6, 298 K): δ (ppm) = 7.35 – 7.31 (m, 1H, Ar), 7.27 – 7.23 (m, 1H, Ar), 7.18 (br, 1H, Ar), 7.13 (d, J = 7.6 Hz, 1H, Ar), 7.06 (s, 4H, Mes-*m*-C*H*), 7.02 – 6.96 (m, 7H, one H from DippNC), 6.94 – 6.87 (m, 3H, Ar), 6.85 (d, J = 7.7 Hz, 2H, DippNC), 3.37 (hept, J = 6.9 Hz, 2H, DippNC), 2.63 (s, 12H, Mes-*o*-CH_3), 2.32 (s, 6H, Mes-*p*-CH_3), 1.19 (s, 18H, $C(CH_3)_3$), 1.06 (d, J = 6.8 Hz, 12H, DippNC), 0.88 (s, 18H, $C(CH_3)_3$).

$^{13}C\{^1H\}$ NMR (100 MHz, C_6D_6, 298 K): δ (ppm) = 175.1 (N*C*N), 172.1 (DippNC), 163.2 (N*C*N), 148.2 (*C*q), 145.2 (DippNC), 143.3 (Mes-*o*-C_q), 137.7 (Mes, C_{ipso}), 134.9 (C_q), 134.3 (C_q), 134.1 (Mes-*p*-C_q), 131.7 (C_q), 130.2, 130.05, 129.99, 129.6, 129.45 (DippNC), 128.6, 128.5 (Mes-*m*-*C*H), 127.98, 127.94, 123.5 (DippNC), 114.3, 53.8 ($C(CH_3)_3$), 53.0 ($C(CH_3)_3$), 32.1 ($C(CH_3)_3$), 31.0 ($C(CH_3)_3$), 30.1 (DippNC), 22.5 (DippNC), 22.0 (Mes-*o*-CH_3), 21.3 (Mes-*p*-CH_3).

$^{29}Si\{^1H\}$ NMR (79.5 MHz, C_6D_6, 298 K): δ (ppm) = –22.1, –105.2.

The NMR signals of DippNC from the mixture are listed below and are comparable to the reported values.[4-5] For comparation, we also measured the NMR spectra of DippNC and obtained consistent data (Figure S7 and S8).

DippN≡C

^1H NMR (400 MHz, C_6D_6, 298 K): δ (ppm) = 7.02 – 6.96 (m, 1H, *p*-C_6H_3), 6.85 (d, J = 7.7 Hz, 2H, *m*-C_6H_3), 3.37 (hept, J = 6.9 Hz, 2H, $CH(CH_3)_2$), 1.06 (d, J = 6.8 Hz, 12H, $CH(CH_3)_2$)

$^{13}C\{^1H\}$ NMR (100 MHz, C_6D_6, 298 K): δ (ppm) = 172.1 (N≡*C*), 145.2 (Ar-*o*-*C*), 129.5 (Ar-*p*-*C*), 123.5 (Ar-*m*-*C*), 30.1 ($CH(CH_3)_2$), 22.5 ($CH(CH_3)_2$). The C_{ipso} is not observed, which differs from

the literature but is consistent with our measurements of DippN≡C in C_6D_6 (Figure S8).

1.4 Synthesis of 3

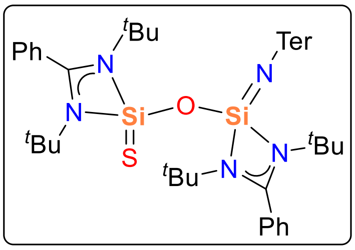

The initial procedures followed those used for the synthesis of compound **2**, with toluene as the solvent. Upon completion of the reaction between CO and compound **1** (0.100 g, 0.095 mmol), as indicated by a color change to yellow, S_8 (3 mg, 0.012 mmol) in toluene (2 mL) was added to the resulting solution containing a mixture of compound **2** and DippNC at room temperature. The solution was stirred for 1 h. All volatiles were then removed under reduced pressure, and the resulting sticky residue was dissolved in a mixture of diethyl ether (0.05 mL) and *n*-pentane (2 mL). The solution was stored at 5 °C for two days, yielding light yellow plate crystals of the target compound.

Yield (based on crystals): 0.052 g (0.379 mmol), 61%.

Anal. Calcd. for $C_{54}H_{71}N_5OSSi_2$ (894.43 g/mol): C, 72.52; H, 8.00; N, 7.83; S, 3.58. Found: C, 72.10; H, 7.95; N, 7.97; S, 3.77.

^{1}H NMR (400 MHz, C_6D_6, 298 K): δ (ppm) = 8.03 – 7.97 (m, 1H), 7.53 – 7.47 (m, 1H), 7.13 – 7.10 (m, 3H), 7.02 (s, 4H, Mes-*m*-C*H*), 6.97 (br, 1H), 6.95 – 6.93 (m, 3H), 6.91 – 6.88 (m, 3H), 6.85 – 6.80 (m, 1H), 2.59 (s, 12H, Mes-*o*-CH_3), 2.30 (s, 6H, Mes-*p*-CH_3), 1.25 (s, 18H, C(CH_3)$_3$), 0.98 (s, 18H, C(CH_3)$_3$).

^{13}C{^{1}H} NMR (100 MHz, C_6D_6, 298 K): δ (ppm) = 176.9 (N*C*N), 175.2 (N*C*N), 148.1 (C_q), 143.5 (Mes-*o*-C_q), 137.6 (Mes, C_{ipso}), 135.3 (C_q), 134.3 (Mes-*p*-C_q), 131.0 (C_q), 130.6 (Ar-*C*H), 130.6 (C_q), 130.3 (Ar-*C*H), 130.2 (Ar-*C*H), 128.6 (Ar-*C*H), 128.4 (Ar-*C*H, from ^{13}C DEPF), 128.3 (Mes-*m*-*C*H, from ^{13}C DEPT), 128.14 (Ar-*C*H, from ^{13}C DEPT), 128.08 (Ar-*C*H, from ^{13}C DEPT), 127.9 (Ar-*C*H, from ^{13}C DEPT), 127.6 (Ar-*C*H), 127.5 (Ar-*C*H), 115.3 (Ar-*C*H), 55.2 (*C*(CH$_3$)$_3$), 54.2 (*C*(CH$_3$)$_3$), 31.6 (C(*C*H$_3$)$_3$), 31.3 (C(*C*H$_3$)$_3$), 21.8 (Mes-*o*-*C*H$_3$), 21.3 (Mes-*p*-*C*H$_3$).

^{29}Si{^{1}H} NMR (79.5 MHz, C_6D_6, 298 K): δ (ppm) = –36.1, –107.8.

IR (ATR): $\tilde{\nu}$ (cm^{-1}) = 2972 (s), 2915 (m), 2871 (w), 1646 (w), 1610 (w), 1577 (w), 1475 (vs), 1445 (s), 1409 vs, 1365 (s), 1284 (w), 1228 (w), 1203 (m), 1109 (w), 1060 (s), 1018 (s), 927 (w), 847 (w), 785 (m), 771 (m), 752 (m), 740 (m), 706 (m), 629 (m), 564 (w), 501 (w), 428 (w).

1.5 Synthesis of 4

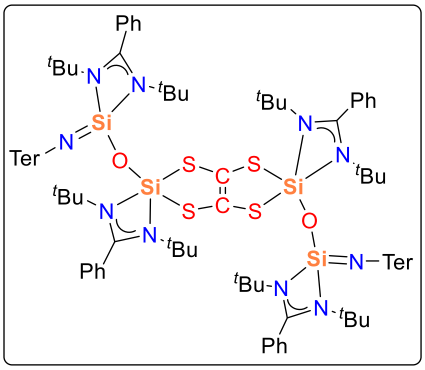

The initial procedures followed those used for the synthesis of compound **2**, with toluene as the solvent. Upon completion of the reaction between CO and compound **1** (0.100 g, 0.095 mmol), as indicated by a color change to yellow, CS_2 (0.007 g, 0.095 mmol) was added to the resulting solution—containing a mixture of compound **2** and DippNC—at room temperature via microsyringe. The color of the solution changed from dark green to dark orange over time. The mixture was stirred for 4 h. All

volatiles were then removed under reduced pressure, and the residue was extracted with diethyl ether. The resulting solution was concentrated and stored at –15 °C for one day, yielding orange plate-like crystals of compound **4**. However, during this reaction, compound **3** consistently formed and co-crystallized with compound **4**. As a result, minor signals corresponding to compound **3** appear in the ^{1}H NMR spectrum. Additionally, compound **4** is not stable in solution and gradually converts to compound **3** over time. This transformation is accelerated by heating at 80 °C in THF, completing within 2 h.

Yield (based on crystals): around 0.035 g (0.018 mmol, except for around 18% of compound **3**, due to unavoidable co-crystallization or decompose), 39%.

Anal. Calcd. for $C_{110}H_{142}N_{10}O_2S_4Si_4$ (1876.99 g/mol): C, 70.39; H, 7.63; N, 7.46; S, 6.83. No suitable data were obtained due to the exist of compound **3**.

^{1}H NMR (400 MHz, C_6D_6, 298 K): δ (ppm) = 8.02 (d, J = 7.9 Hz, 1H), 7.26 (d, J = 7.5 Hz, 1H), 7.13 (d, J = 7.3 Hz, 3H), 7.08 (d, J = 9.3 Hz, 1H), 7.02 (br, 1H), 6.97 (s, 4H, Mes-*m*-C*H*), 6.94 (d, J = 7.7 Hz, 1H), 6.92 – 6.85 (m, 5H, 1H from compound **3** due to the overlaps), 6.79 (t, J = 7.5 Hz, 1H), 2.54 (s, 12H, Mes-*o*-CH_3), 2.26 (s, 6H, Mes-*p*-CH_3), 1.27 (s, 18H, C(CH_3)$_3$), 0.83 (s, 18H, C(CH_3)$_3$).

^{13}C{^{1}H} NMR (100 MHz, C_6D_6, 298 K): δ (ppm) = 182.2 (N*C*N), 177.9 (N*C*N), 147.2 (C_q), 143.1 (Mes-*o*-C_q), 137.3 (Mes, C_{ipso}), 135.0 (C_q), 134.6 (C_q), 131.2 (Ar-*C*H), 130.5 (Ar-*C*H), 130.34 (Ar-*C*H), 130.27 (Ar-*C*H), 130.0 (Ar-*C*H), 129.0 (C_q), 128.74 (Ar-*C*H), 128.70 (C_q), 128.6 (Mes-*m*-*C*H), 128.4 (Ar-*C*H), 128.2 (Ar-*C*H), 127.4 (Ar-*C*H), 115.6 (Ar-*C*H), 56.7 (*C*(CH$_3$)$_3$), 54.1 (*C*(CH$_3$)$_3$), 31.3 (C(*C*H$_3$)$_3$), 31.2 (C(*C*H$_3$)$_3$), 21.9 (Mes-*o*-*C*H$_3$), 21.2 (Mes-*p*-*C*H$_3$). Note: the [CS_2]$_2$ resonance could not be observed.

^{29}Si{^{1}H} NMR (79.5 MHz, C_6D_6, 298 K): δ (ppm) = –107.9, –111.1.

IR (ATR): this was not measured due to the compound **4** is obtained with a small amount of compound **3**.

1.6 Synthesis of 5

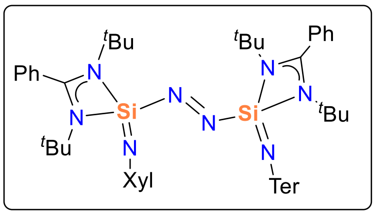

Toluene (10 mL) was added to a mixture of compound **1** (0.100 g, 0.095 mmol) and [XylNC] (0.012 g, 0.095 mmol) at room temperature, and the solution was stirred for 1 h. All volatiles were then removed under reduced pressure, and the resulting sticky residue was dissolved in a mixture of diethyl ether (1 mL) and *n*-pentane (2 mL). The solution was stored at −15 °C for two days, yielding red crystalline material of the target compound. Crystals suitable for XRD measurement were grown from a concentrated C_6D_6 solution of compound **5**.

Yield (based on crystals): 0.060 g (0.064 mmol), 68%.

Anal. Calcd. for $C_{62}H_{80}N_8Si_2$ (993.55 g/mol): C, 74.95; H, 8.12; N, 11.28. Found: C, 75.27; H, 8.10; N, 11.17.

^{1}H NMR (400 MHz, C_6D_6, 298 K): δ (ppm) = 7.45 (d, J = 7.3 Hz, 2H, Xyl), 7.32 – 7.27 (m, 1H), 7.21 (d, J = 7.3 Hz, 3H), 7.10 – 7.04 (m, 2H), 7.01 (d, J = 7.0 Hz, 1H), 6.96 – 6.92 (m, 7H, 4H from Mes), 6.92 – 6.88 (m, 4H), 2.72 (s, 6H, Xyl-Me), 2.58 (s, 12H, Mes-*o*-CH_3), 2.24 (s, 6H, Mes-*p*-

CH_3), 1.09 (s, 18H, C(CH_3)$_3$), 0.70 (s, 18H, C(CH_3)$_3$).

^{13}C{^{1}H} NMR (100 MHz, C_6D_6, 298 K): δ (ppm) = 178.1 (N*C*N), 177.4 (N*C*N), 150.6 (Xyl-C_q), 147.3 (C_q), 142.8 (Mes-*o*-C_q), 137.4 (Mes, C_{ipso}), 135.0 (C_q), 134.5 (Mes-*p*-C_q), 130.7, 130.4, 130.3 (C_q), 130.2, 130.0 (C_q), 129.4 (Xyl-*p*-*C*H), 128.7 (C_q), 128.5, 128.4, 128.3 (from ^{13}C DEPT), 128.2 (Mes-*m*-*C*H, from ^{13}C DEPT), 128.0 (from ^{13}C DEPT), 127.92, 127.86 (Xyl-*m*-*C*H, from ^{13}C DEPT), 116.0, 115.7, 54.5 (*C*(CH_3)$_3$), 53.8 (*C*(CH_3)$_3$), 31.4 (C(CH_3)$_3$), 30.8 (C(CH_3)$_3$), 22.1 (Xyl-CH_3), 21.6 (Mes-*o*-CH_3), 21.2 (Mes-*p*-CH_3).

^{29}Si{^{1}H} NMR (79.5 MHz, C_6D_6, 298 K): δ (ppm) = –99.1, –108.3.

IR (ATR): $\tilde{\nu}$ (cm^{-1}) = 2967 (m), 2913 (m), 2867 (w), 1515 (s), 1475 (m), 1449 (m), 1398 (vs), 1364 (s), 1283 (w), 1222 (w), 1200 (m), 1096 (w), 1075 (sh), 1025 (w), 923 (w), 902 (w), 850 (w), 792 (w), 755 (m), 705 (w), 683 (w), 646 (w), 504 (w), 490 (w).

1.7 Synthesis of 6

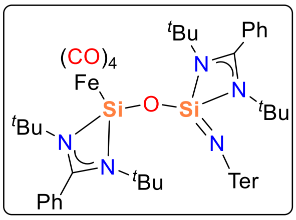

$Fe(CO)_5$ (0.028 g, 0.143 mmol) was added via microsyringe to a toluene solution of compound **1** (0.150 g, 0.143 mmol) at room temperature. The solution color gradually changed from dark red to orange. After stirring overnight, the solution was concentrated, affording colorless crystals surrounded by a sticky residue identified as DippNC. The crystals were isolated by filtration and washed with *n*-pentane to yield the final complex.

Alternative method: After the reaction between CO and compound **1** (0.150 g, 0.143 mmol) was complete, as evidenced by a color change to yellow, $Fe(CO)_5$ (0.028 g, 0.143 mmol) was added via microsyringe. The mixture was stirred overnight, and the solution was then concentrated to afford complex **6**.

Yield (based on crystals): 0.075 – 0.088 g (0.073 – 0.085 mmol), 51% – 60%.

Anal. Calcd. for $C_{58}H_{71}FeN_5O_5Si_2$ (1030.25 g/mol): C, 67.62; H, 6.95; N, 6.80. Found: C, 67.71; H, 7.19; N, 6.84.

^{1}H NMR (400 MHz, THF-d_8, 298 K): δ (ppm) = 7.92 (d, *J* = 7.3 Hz, 1H, Ph), 7.69 – 7.64 (m, 2H, Ph), 7.56 (d, *J* = 8.9 Hz, 1H, Ph), 7.51 – 7.43 (m, 4H, Ph), 7.32 – 7.26 (m, 2H), 6.83 (s, 4H, Mes-*m*-C*H*), 6.54 (d, *J* = 7.8 Hz, 2H, Ter-Ar_{center}-*m*-C*H*), 6.48 – 6.42 (m, 1H, Ter-Ar_{center}-*p*-C*H*), 2.27 (s, 12H, Mes-*o*-CH_3), 2.20 (s, 6H, Mes-*p*-CH_3), 1.28 (s, 18H, C(CH_3)$_3$), 0.72 (s, 18H, C(CH_3)$_3$).

^{13}C{^{1}H} NMR (100 MHz, C_6D_6): δ (ppm) = 217.6 (*C*O), 178.3 (N*C*N), 171.9 (N*C*N), 148.0 (Ter-Ar_{center}-C_q), 144.0 (Mes-*o*-C_q), 137.6 (Mes, C_{ipso}), 135.6 (C_q), 135.1 (Mes-*p*-C_q), 132.3 (Ar-*C*H), 131.6 (Ar-*C*H), 131.1 (C_q), 130.99 (Ter-Ar_{center}-*m*-CH), 130.95 (C_q), 130.1 (Ar-*C*H), 129.7 (Ar-*C*H), 129.5 (Ar-*C*H), 129.4 (Mes-*m*-*C*H), 128.9 (Ar-*C*H), 128.7 (Ar-*C*H), 128.4 (Ar-*C*H), 115.1 (Ter-Ar_{center}-*p*-CH), 56.2 (*C*(CH_3)$_3$), 54.4 (*C*(CH_3)$_3$), 32.1 (C(CH_3)$_3$), 31.4 (C(CH_3)$_3$), 22.3 (Mes-*o*-CH_3), 21.3 (Mes-*p*-CH_3).

^{29}Si{^{1}H} NMR (79.5 MHz, C_6D_6, 298 K): δ (ppm) = –102.8, –109.5.

IR (ATR): $\tilde{\nu}$ (cm^{-1}) = 2972 (w), 2931 (w), 2915 (w), 2026 (s, *CO*), 1944 (s, *CO*), 1914 (vs, *CO*), 1898 (vs, *CO*), 1507 (m), 1454 (w), 1397 (m), 1367 (w), 1280 (w), 1201 (w), 1111 (w), 1060 (m), 1020 (m), 929 (w), 851 (w), 786 (w), 754 (w), 708 (w), 627 (s), 502 (w).

2. NMR spectra

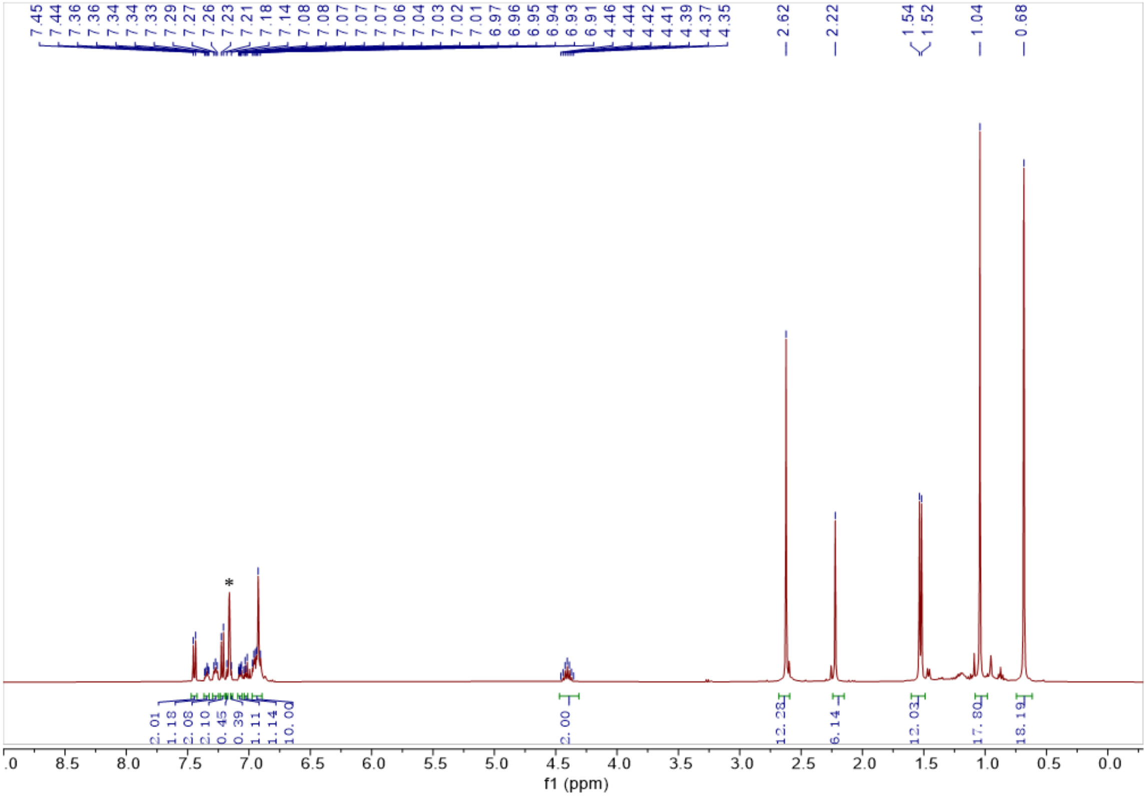

Figure S1. ^{1}H NMR spectrum (400 MHz, 298 K) of **1** in C_6D_6. *, residual protio solvent signal.

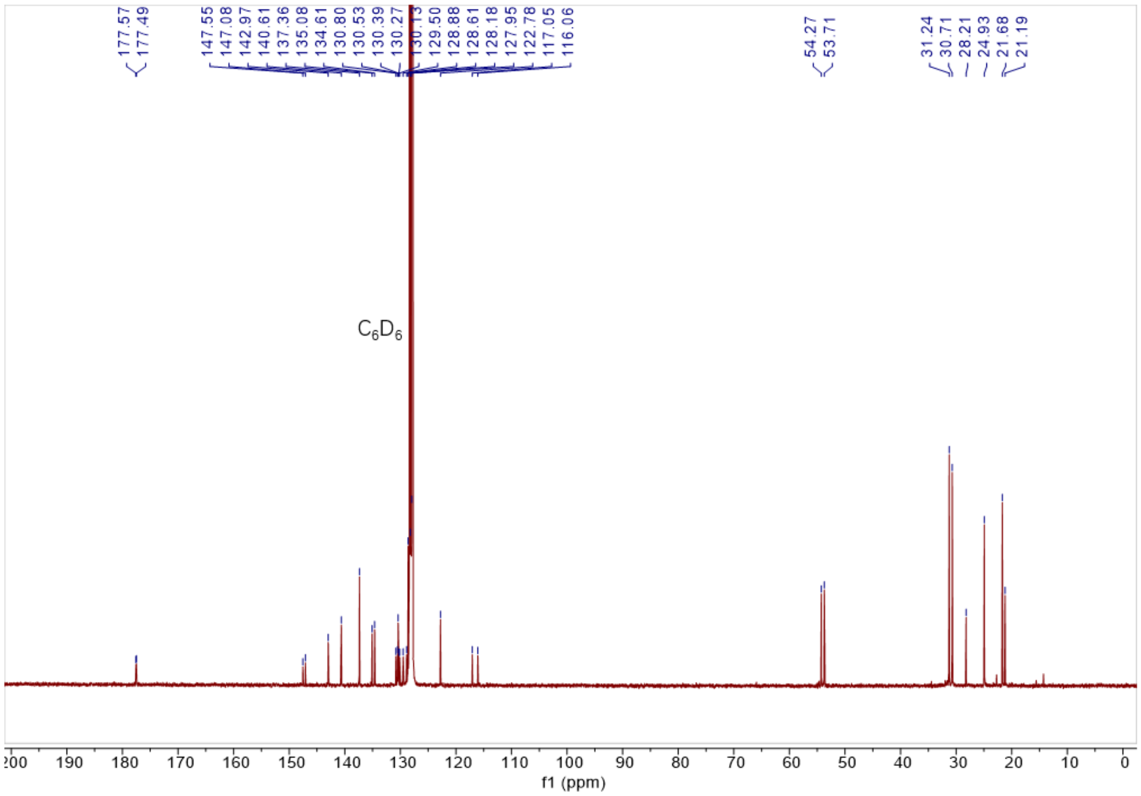

Figure S2. $^{13}C\{^{1}H\}$ NMR spectrum (101 MHz, 298 K) of **1** in C_6D_6.

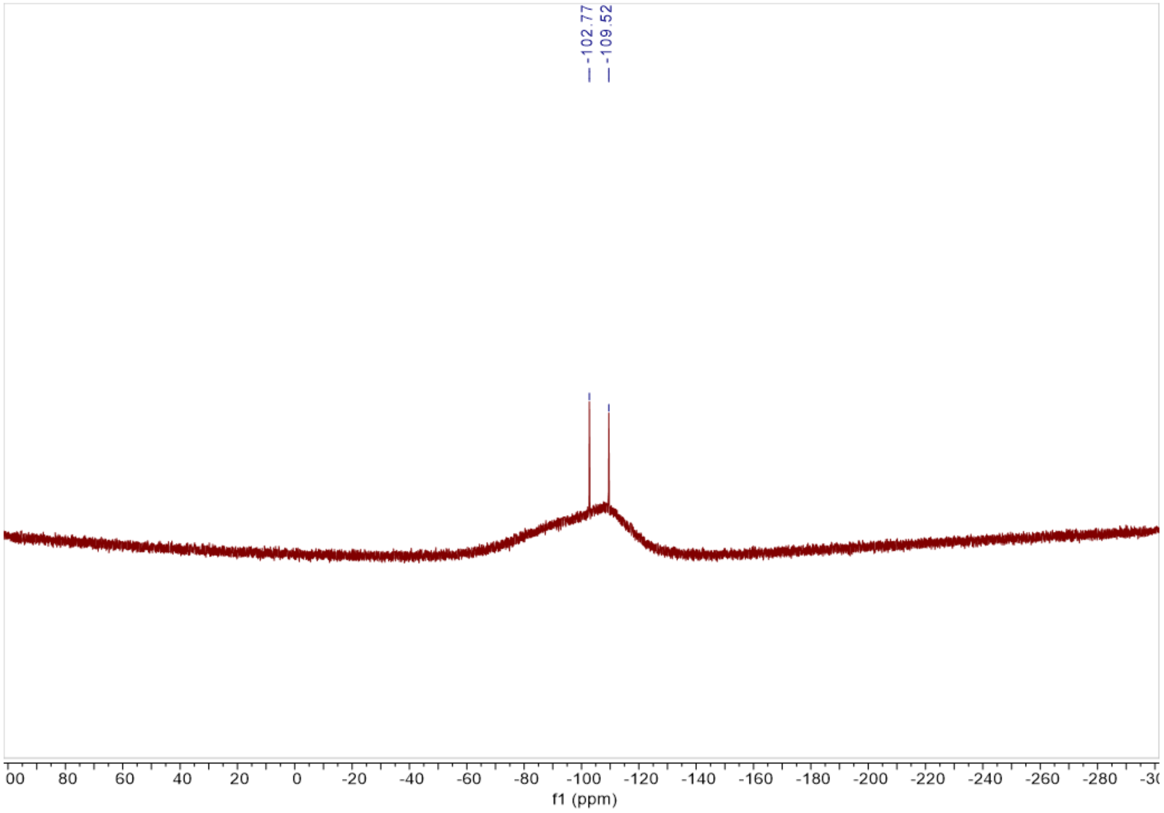

Figure S3. $^{29}Si\{^{1}H\}$ NMR (79.5 MHz, 298 K) spectrum of **1** in C_6D_6.

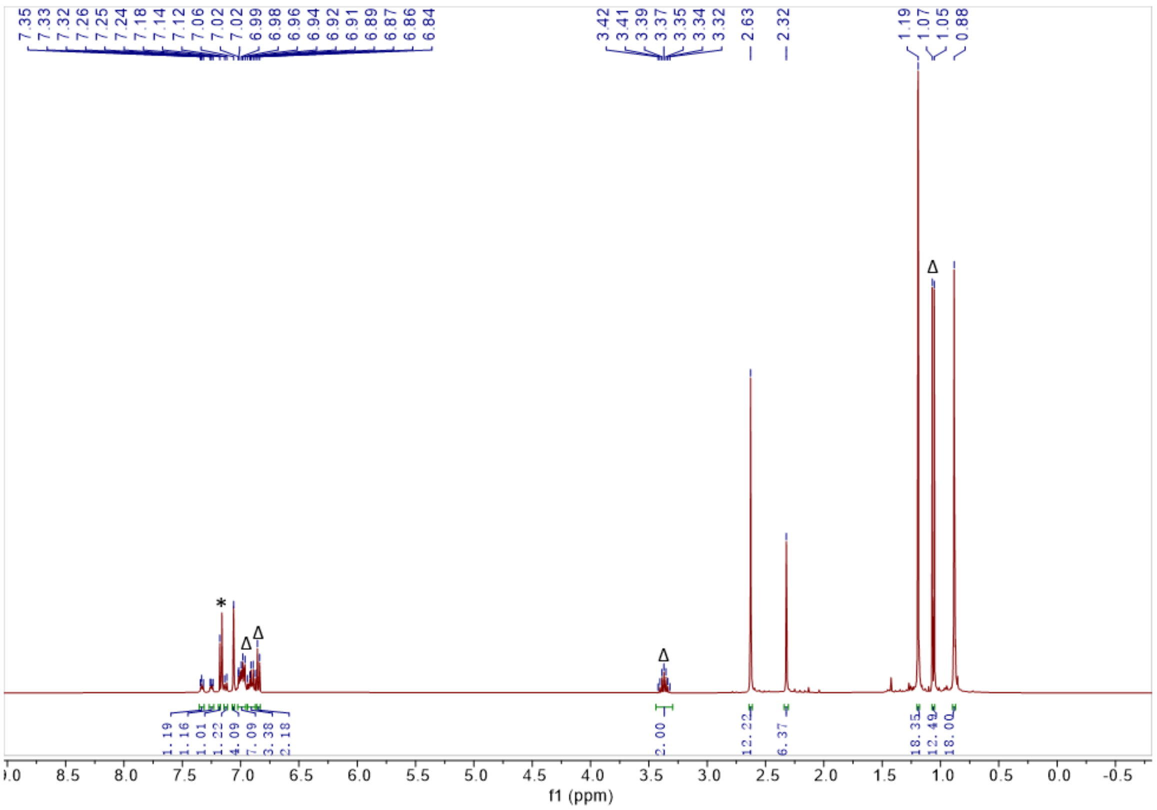

Figure S4. ^{1}H NMR spectrum (400 MHz, 298 K) of **2** with **DippNC** in C_6D_6. *, residual protio solvent signal; Δ, signals of DippNC.

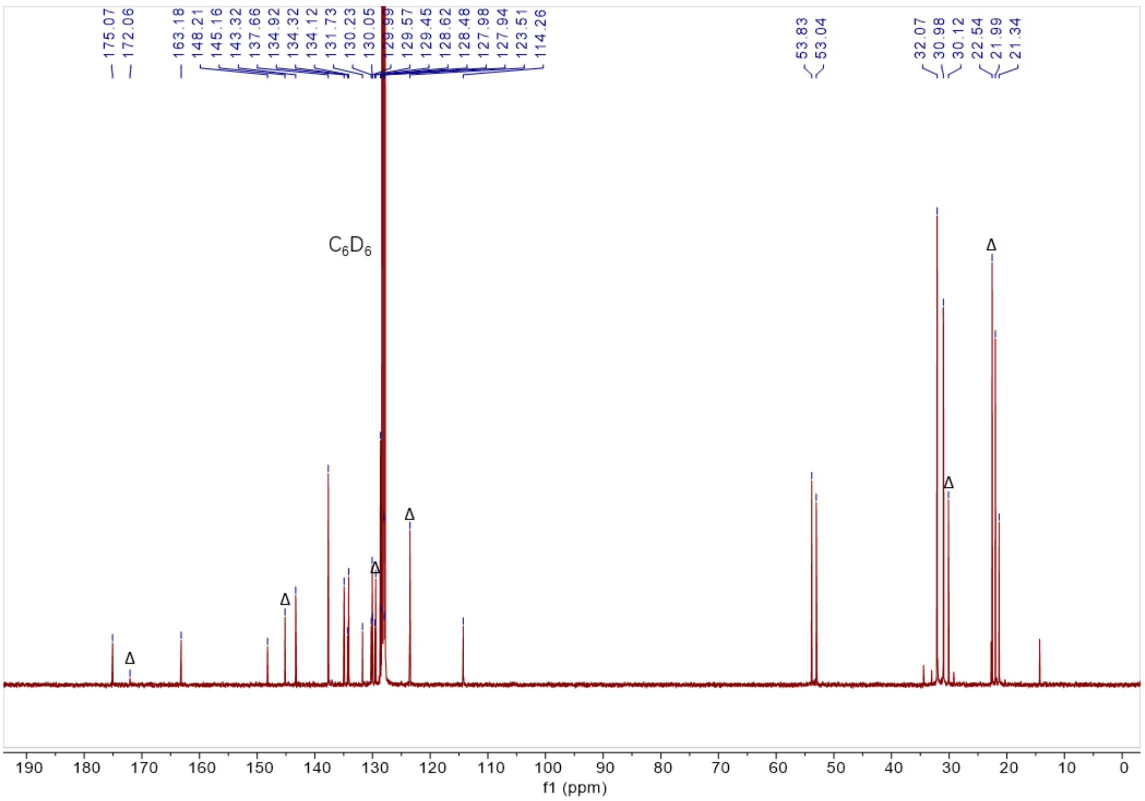

Figure S5. $^{13}C\{^1H\}$ NMR spectrum (101 MHz, 298 K) of **2** with **DippNC** in C_6D_6. Δ, signals of DippNC.

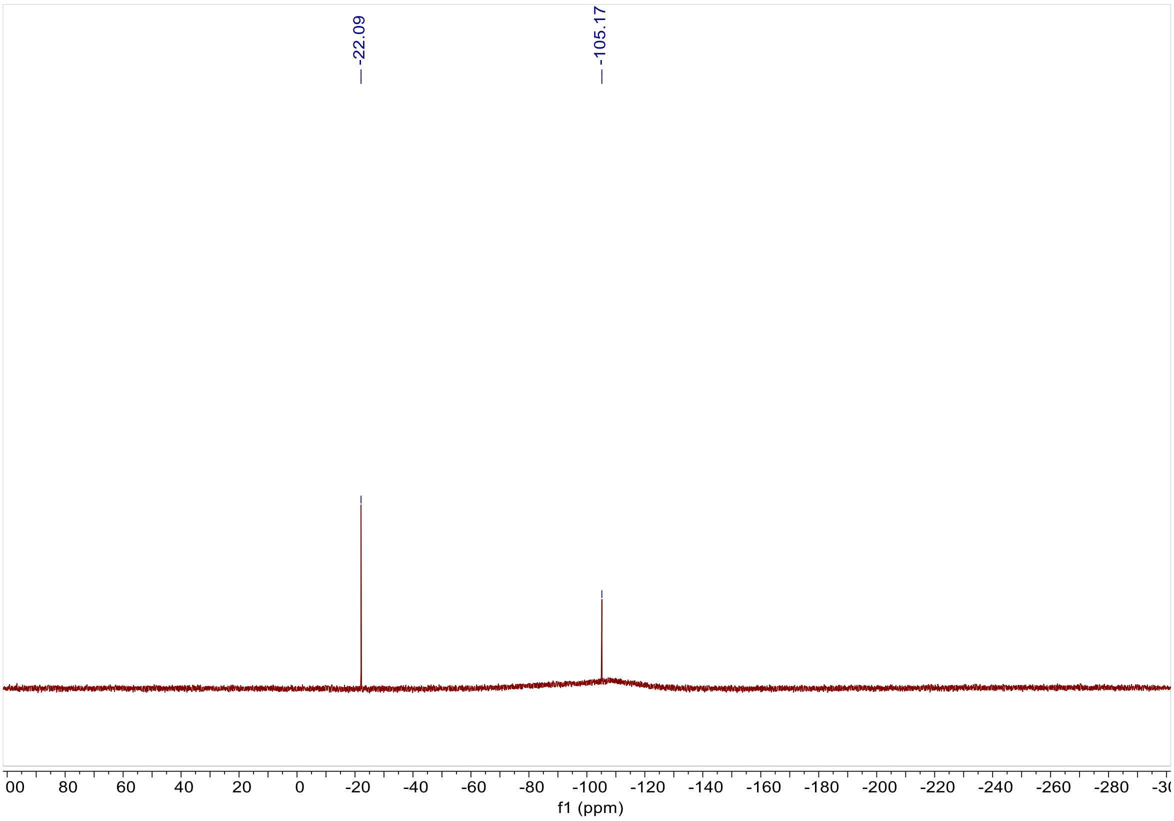

Figure S6. $^{29}Si\{^1H\}$ NMR (79.5 MHz, 298 K) spectrum of **2** (with **DippNC**) in C_6D_6.

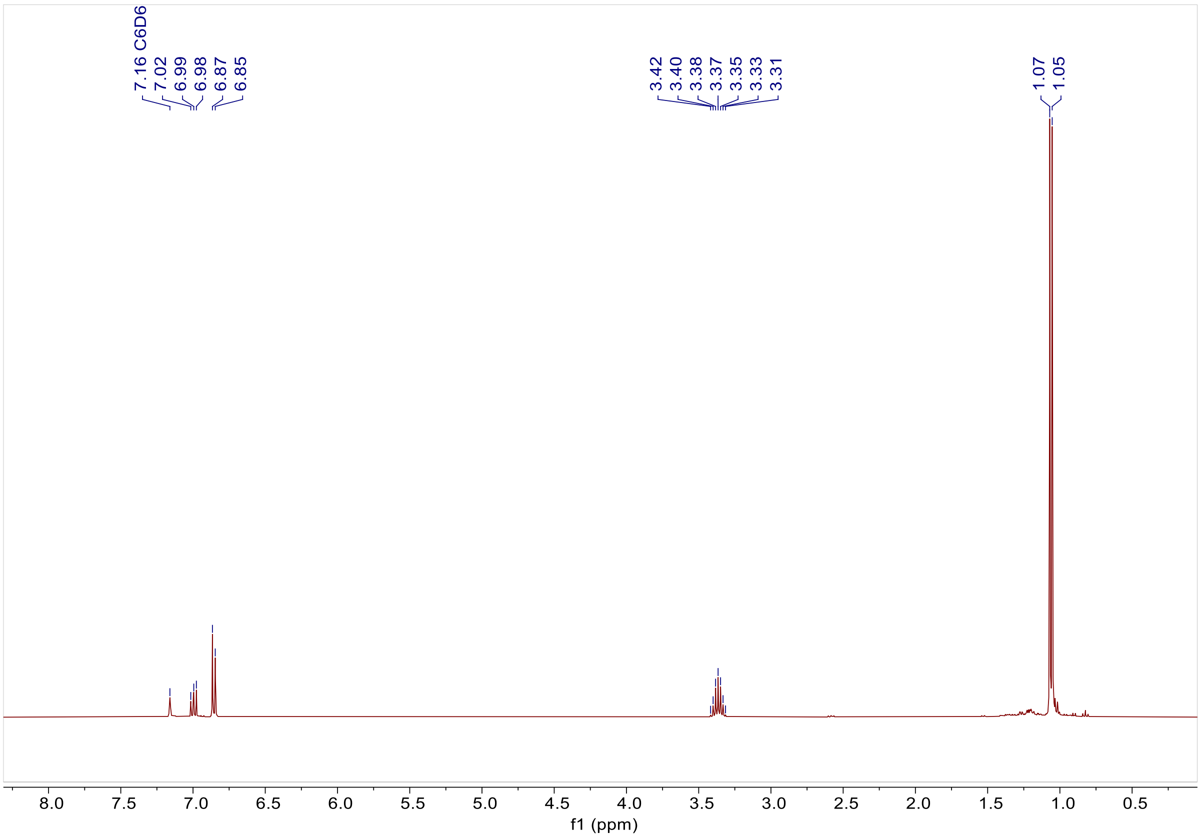

Figure S7. ^{1}H NMR spectrum (400 MHz, 298 K) of **DippNC** in C_6D_6.

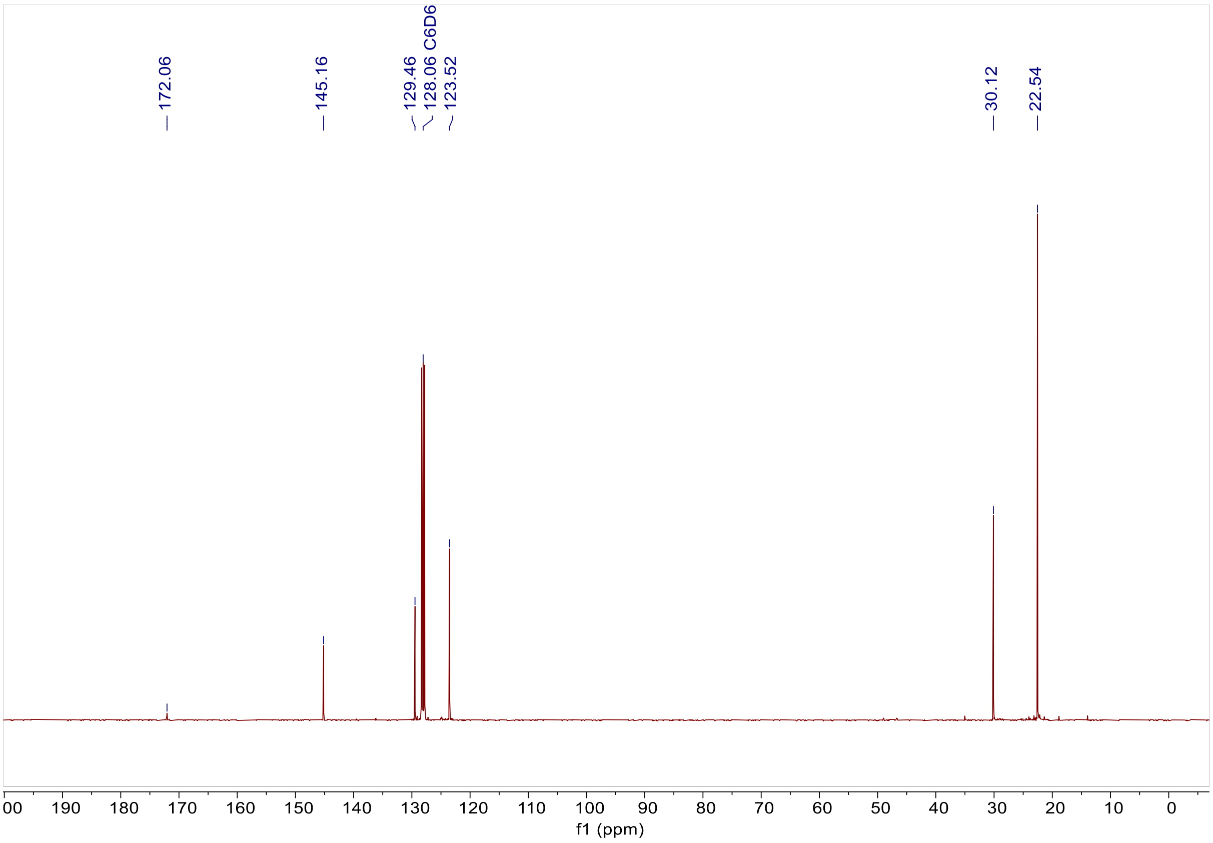

Figure S8. $^{13}C\{^{1}H\}$ NMR spectrum (101 MHz, 298 K) of **DippNC** in C_6D_6.

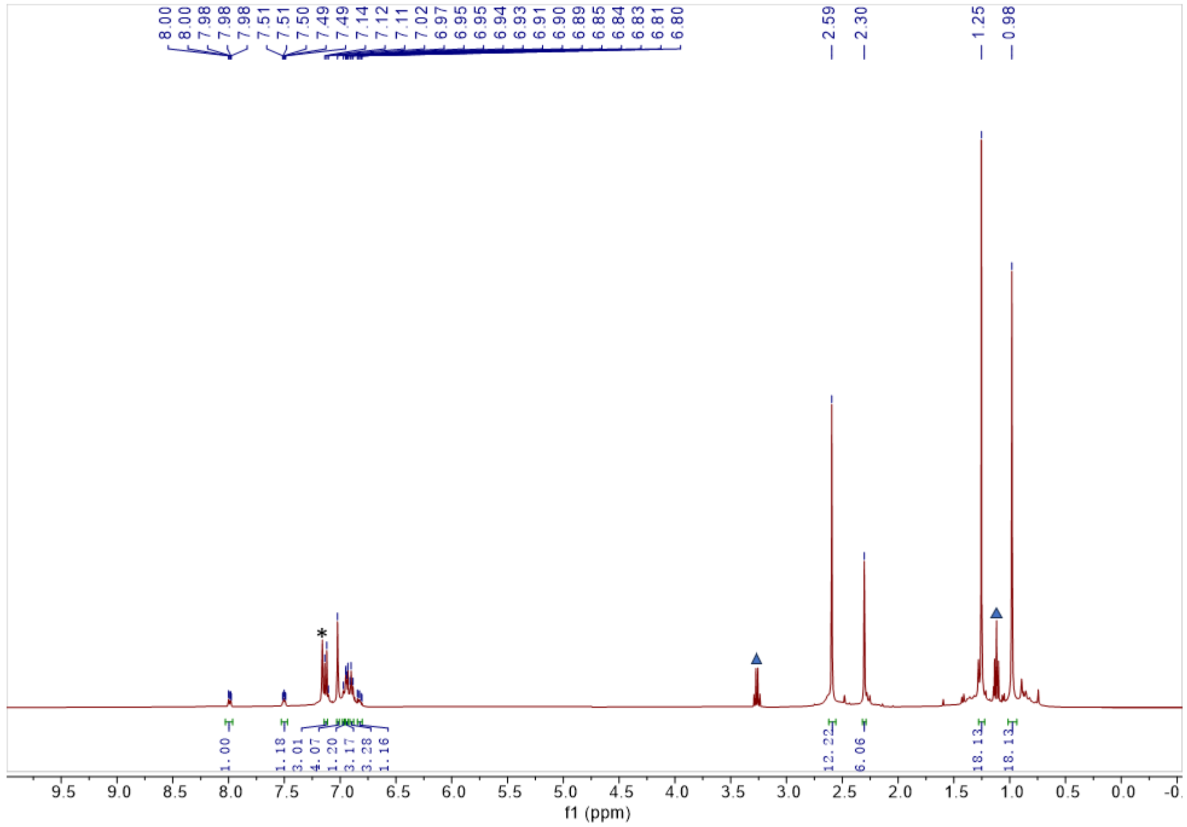

Figure S9. ^{1}H NMR spectrum (400 MHz, 298 K) of **3** in C_6D_6. *, residual protio solvent signal; Δ, signals of Et_2O.

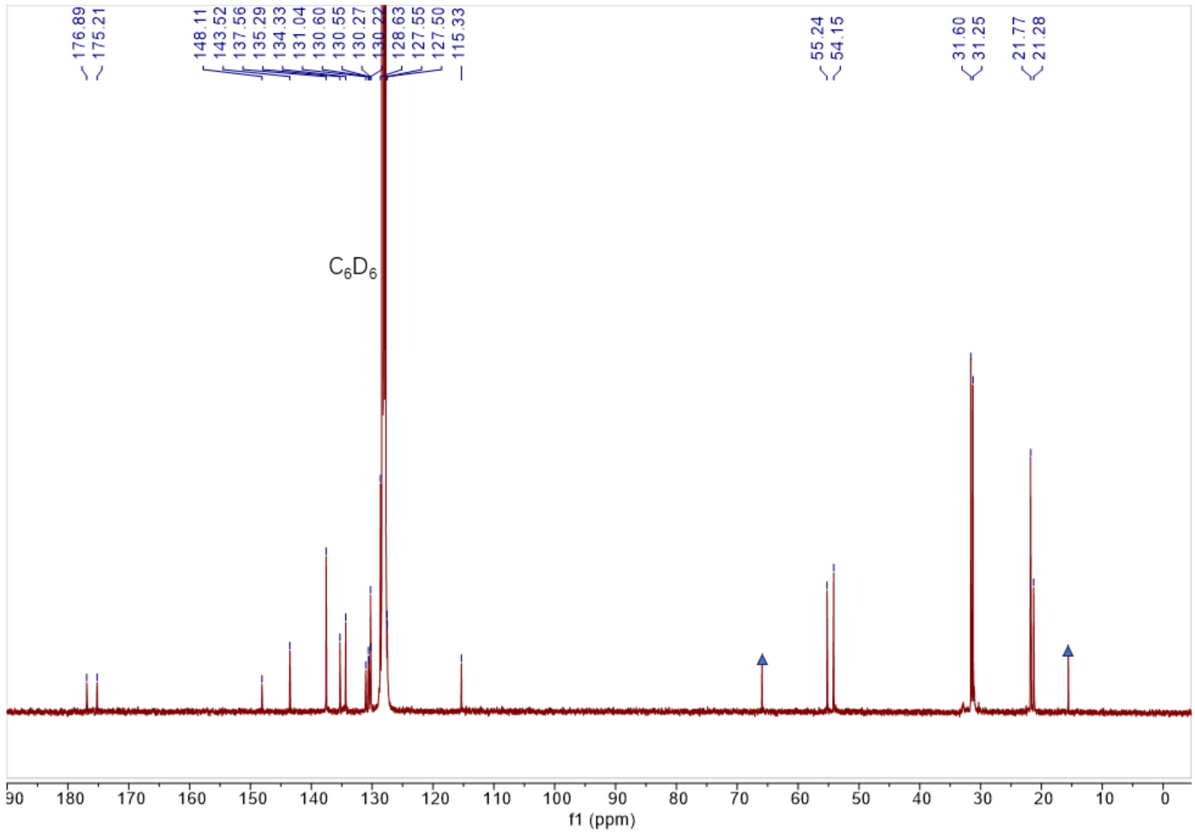

Figure S10. $^{13}C\{^{1}H\}$ NMR spectrum (101 MHz, 298 K) of **3** in C_6D_6. Δ, signals of Et_2O.

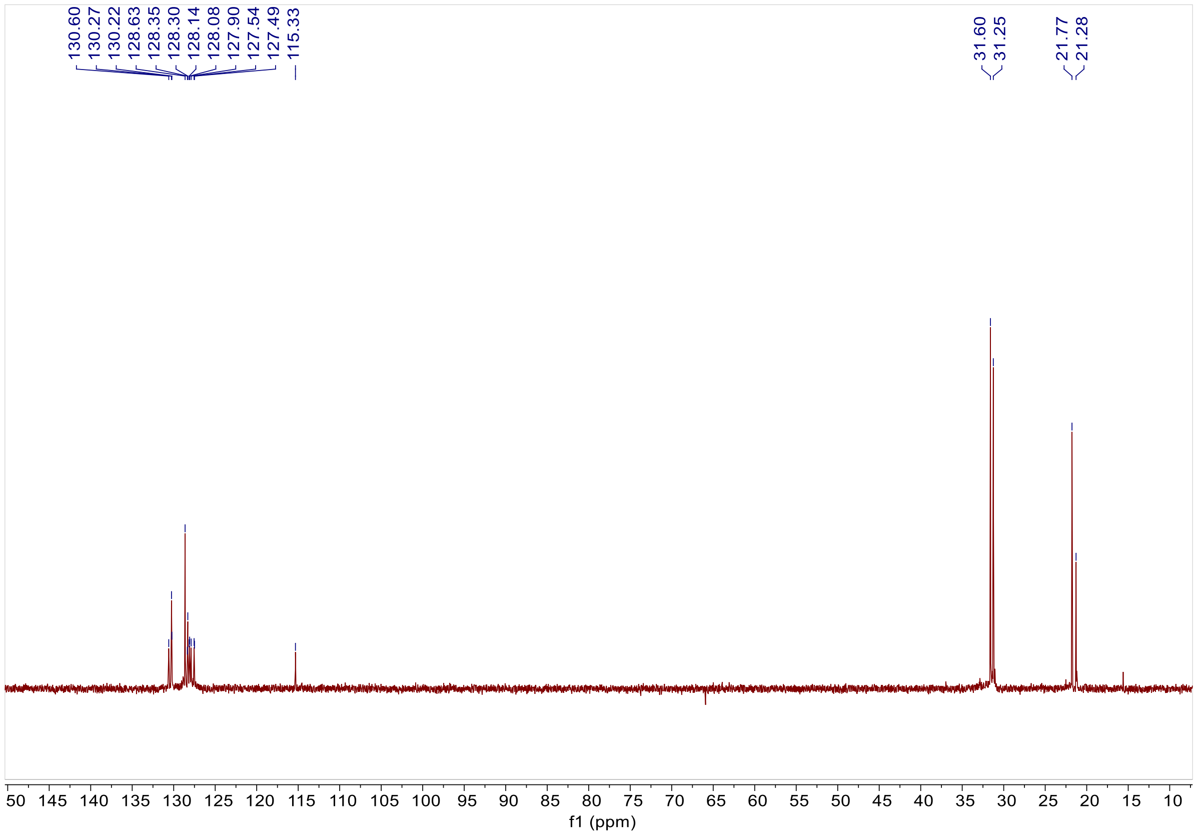

Figure S11. ^{13}C DEPT-135 NMR spectrum (101 MHz, 298 K) of **3** in C_6D_6.

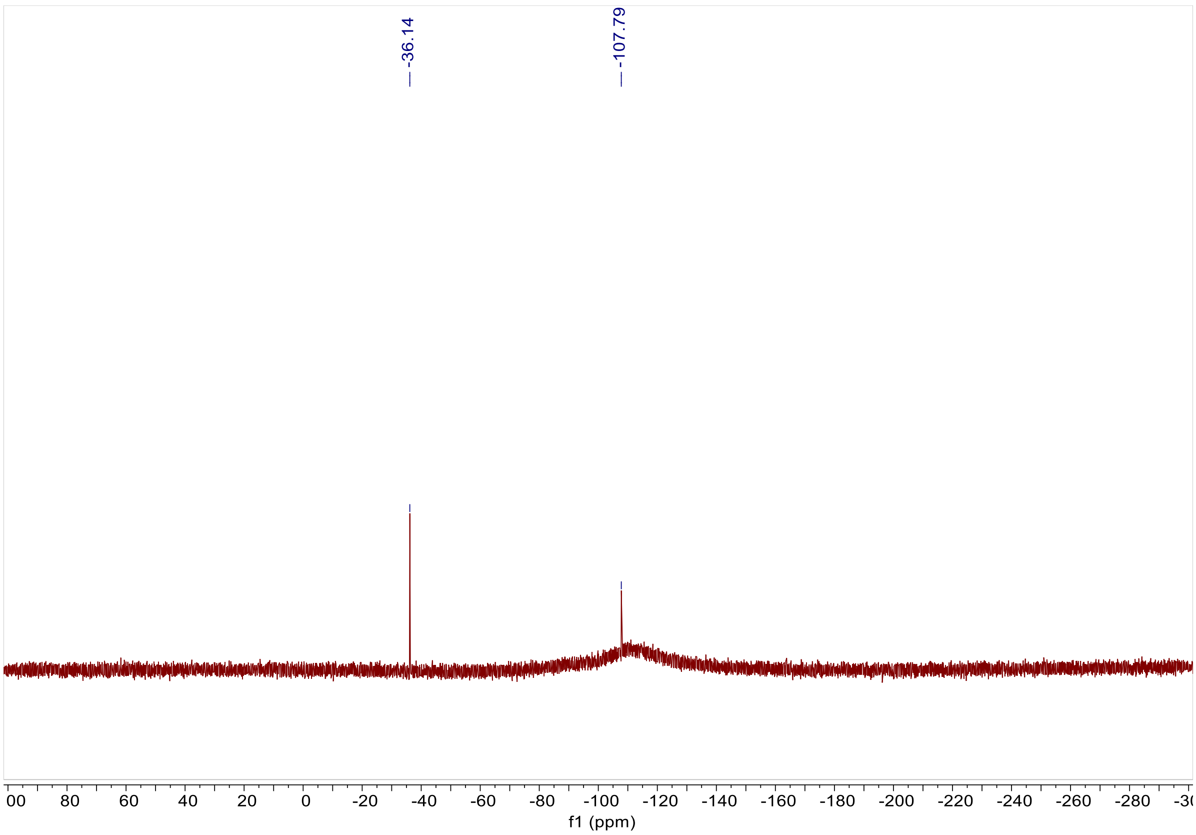

Figure S12. $^{29}Si\{^1H\}$ NMR (79.5 MHz, 298 K) spectrum of **3** in C_6D_6.

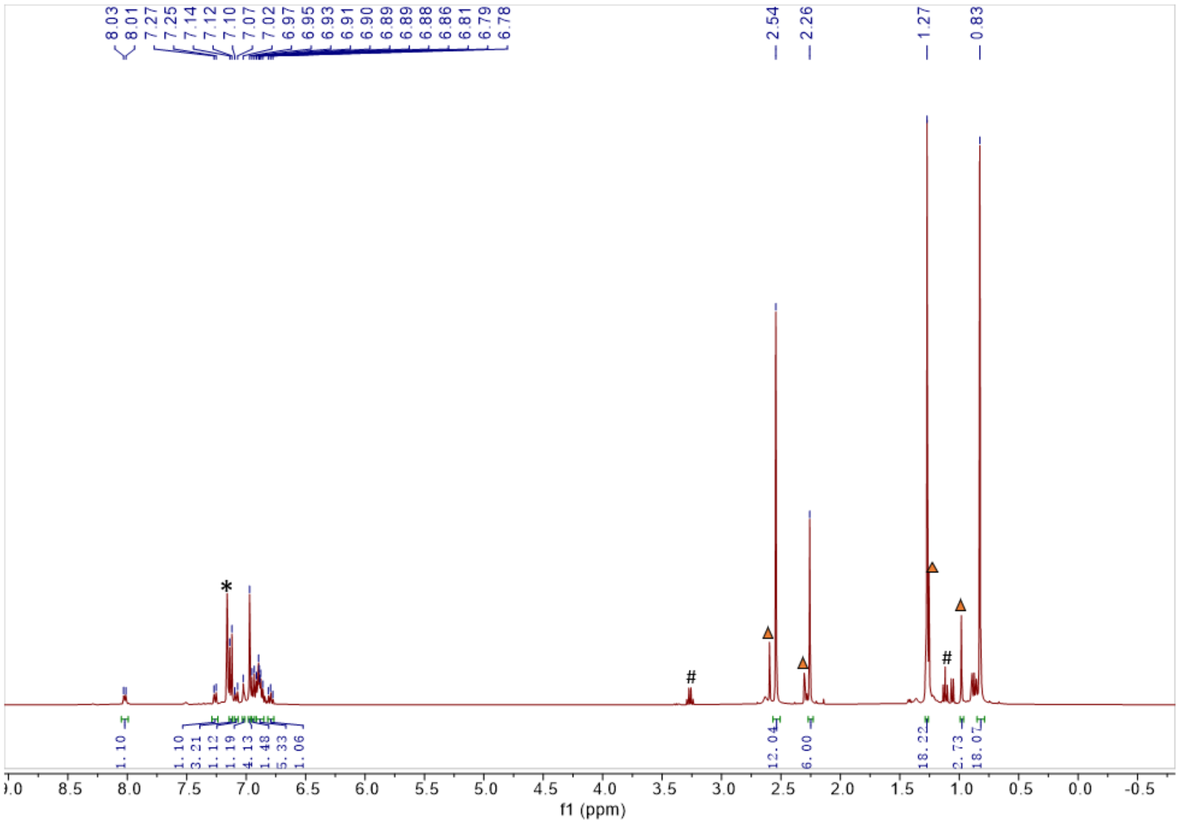

Figure S13. ^{1}H NMR spectrum (400 MHz, 298 K) of **4** (with co-crystallized **3**) in C_6D_6. *, residual protio solvent signal; Δ, signals of compound **3**; #, signals of Et_2O.

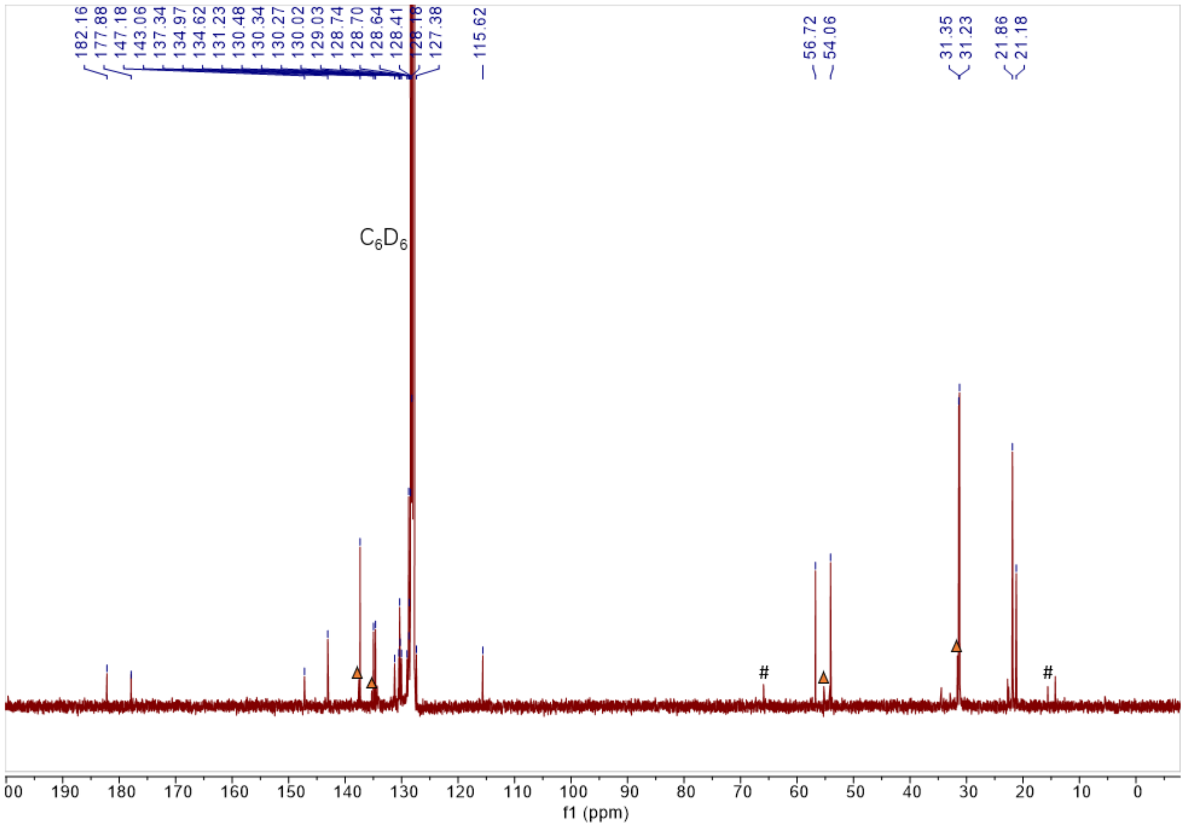

Figure S14. $^{13}C\{^{1}H\}$ NMR spectrum (101 MHz, 298 K) of **4** (with co-crystallized **3**) in C_6D_6. Δ, signals of compound **3**; #, signals of Et_2O.

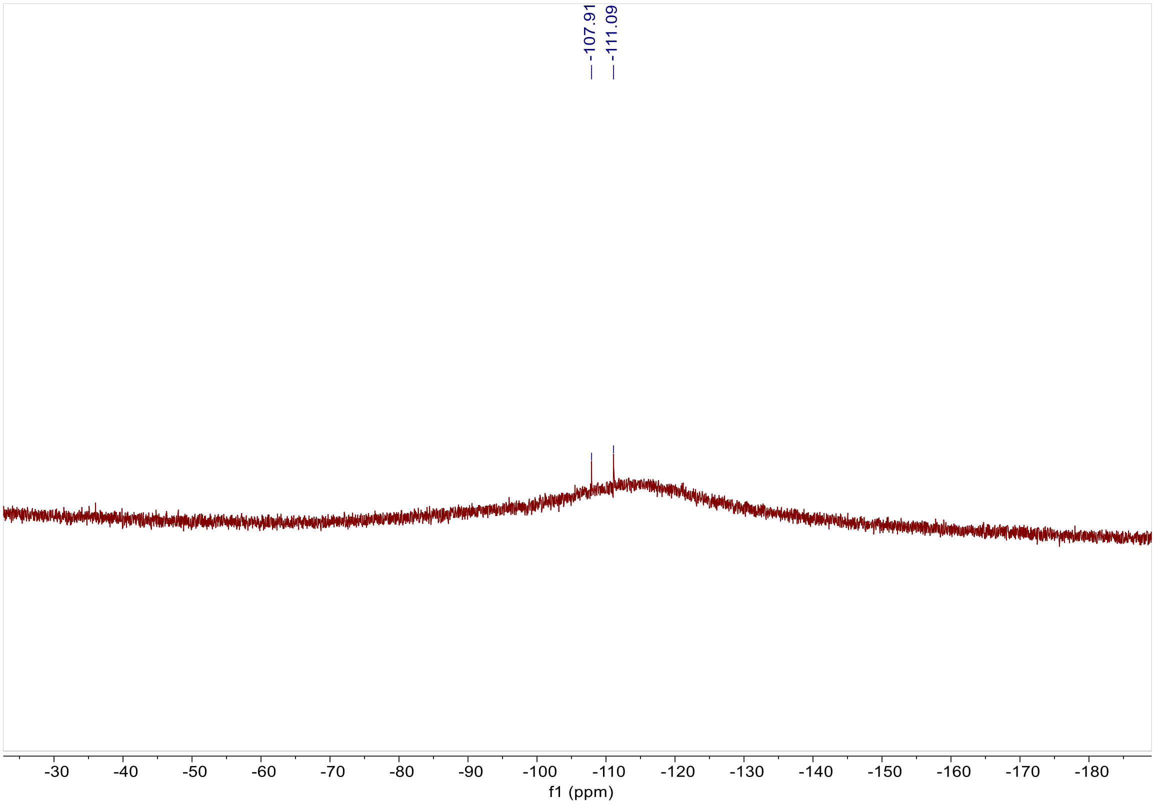

Figure S15. $^{29}Si\{^{1}H\}$ NMR (79.5 MHz, 298 K) spectrum of **4** (with co-crystallized **3**) in C_6D_6. No signals are observed for **3** due to its low concentration.

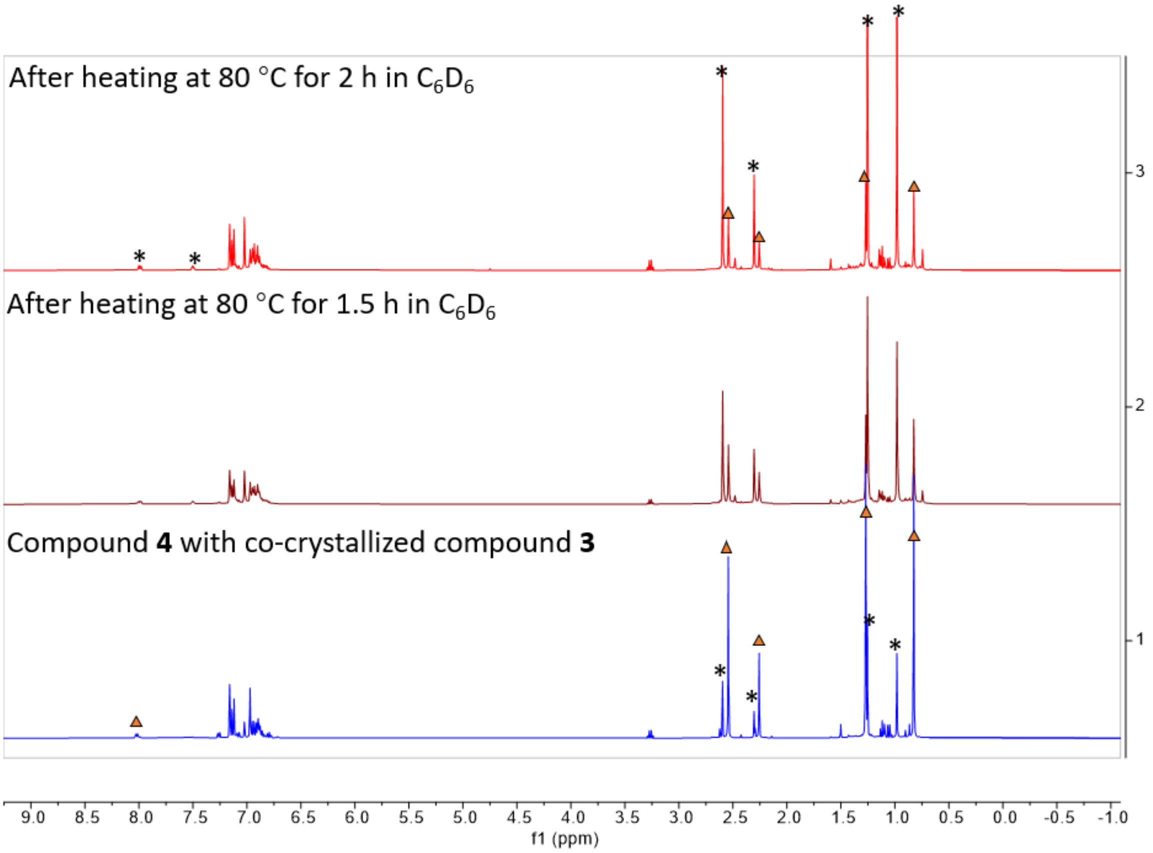

Figure S16. Stacked ^{1}H NMR spectra showing the transformation of compound **4** to compound **3** upon heating. Δ, signals of compound **4**; *, signals of compound **3**.

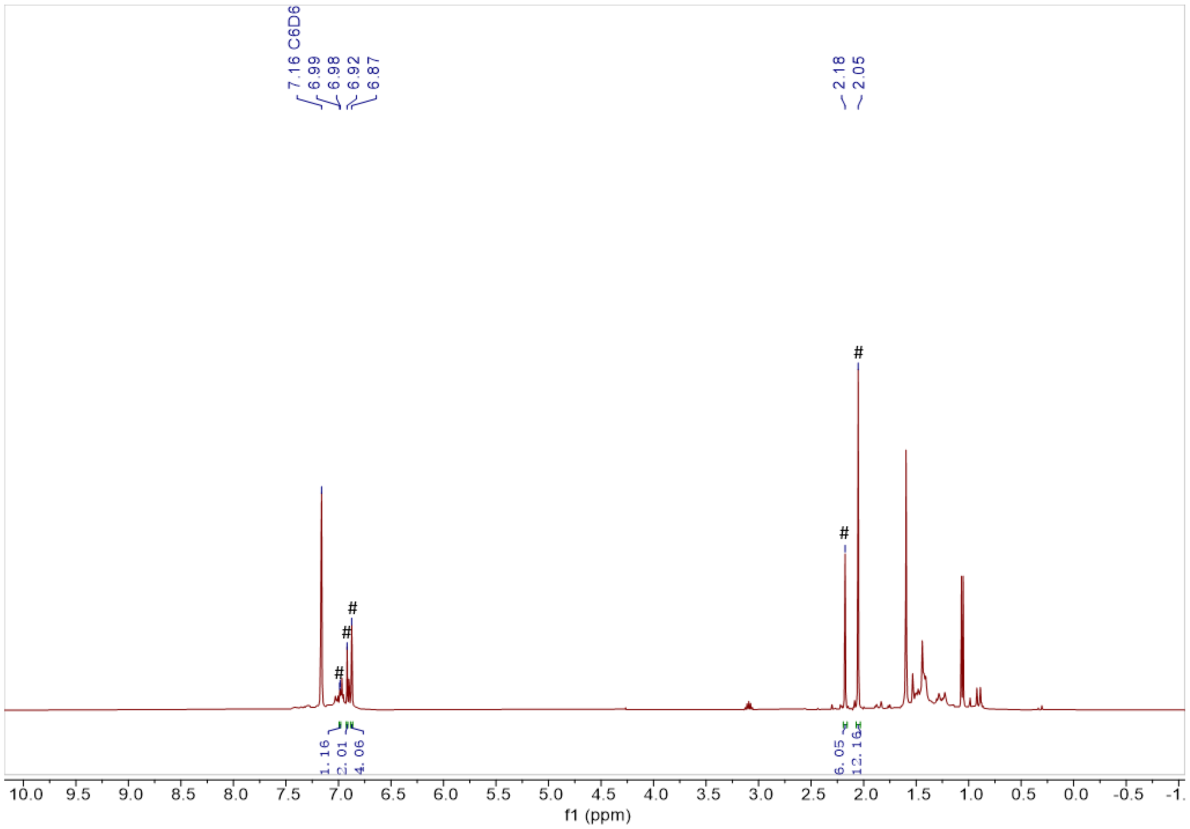

Figure S17. The reaction of compound **1** with CO_2 in C_6D_6. #, signals of the **TerNCO**, consistent with those reported in the literature.[6-7]

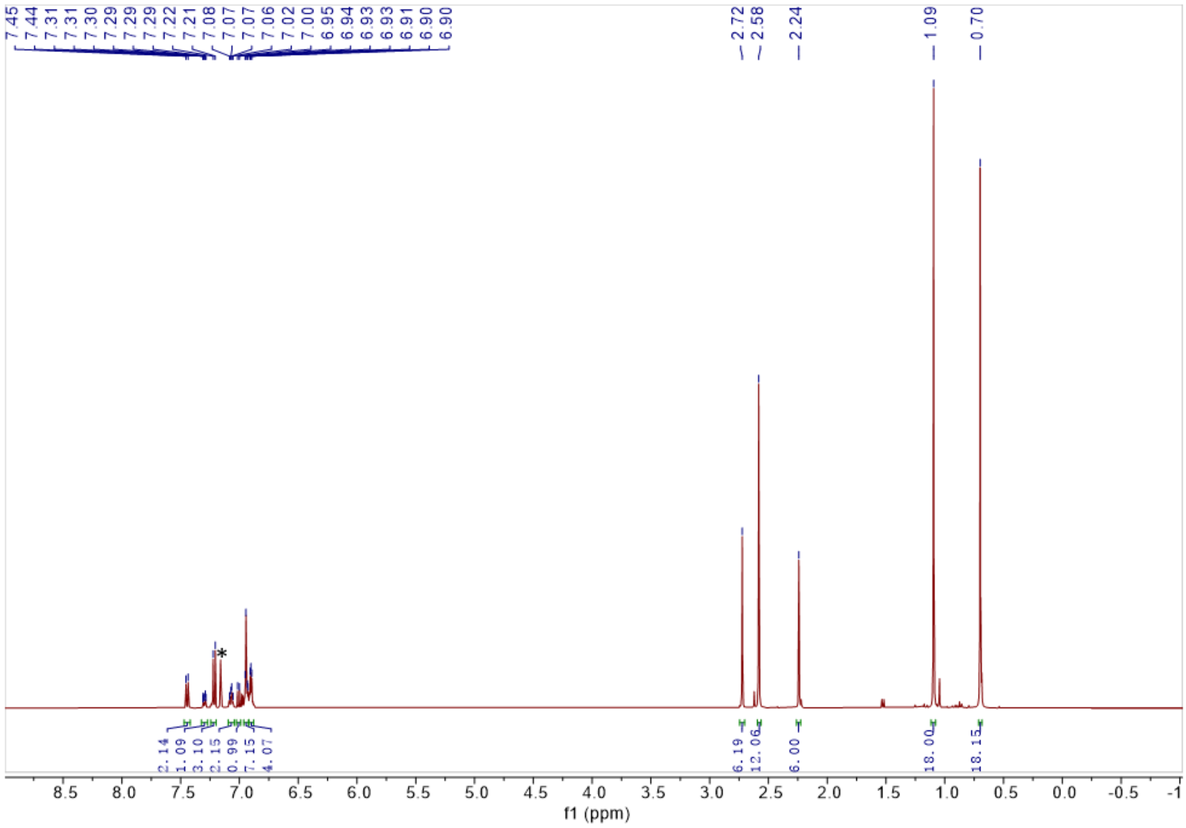

Figure S18. ^1H NMR spectrum (400 MHz, 298 K) of **5** in C_6D_6. *, residual protio solvent signal.

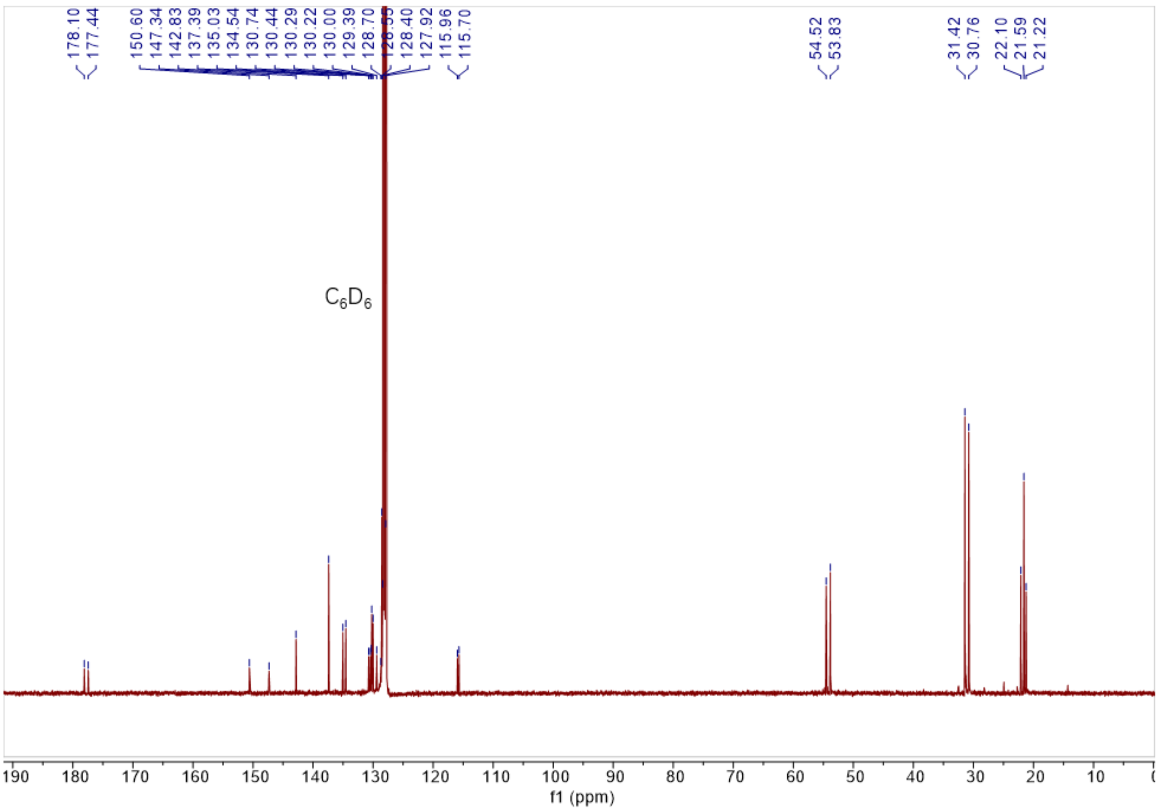

Figure S19. $^{13}C\{^{1}H\}$ NMR spectrum (101 MHz, 298 K) of **5** in C_6D_6.

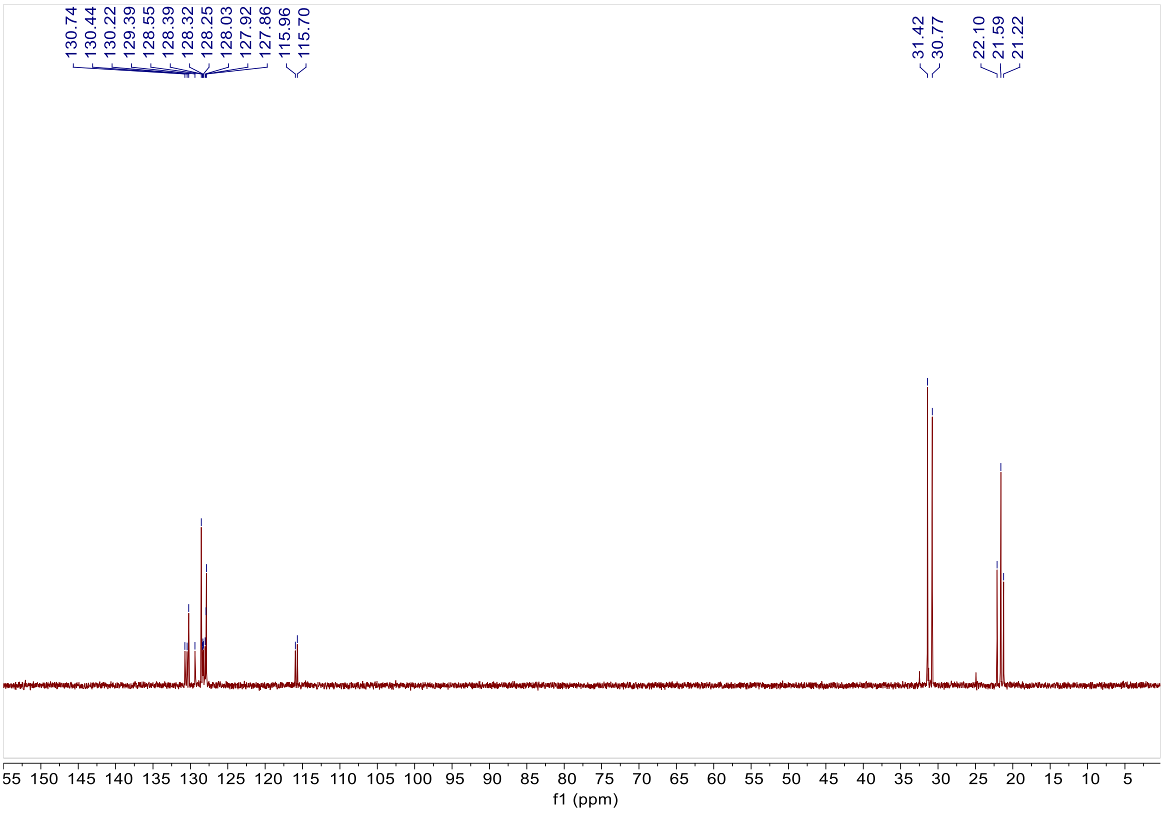

Figure S20. ^{13}C DEPT-135 NMR spectrum (101 MHz, 298 K) of **5** in C_6D_6.

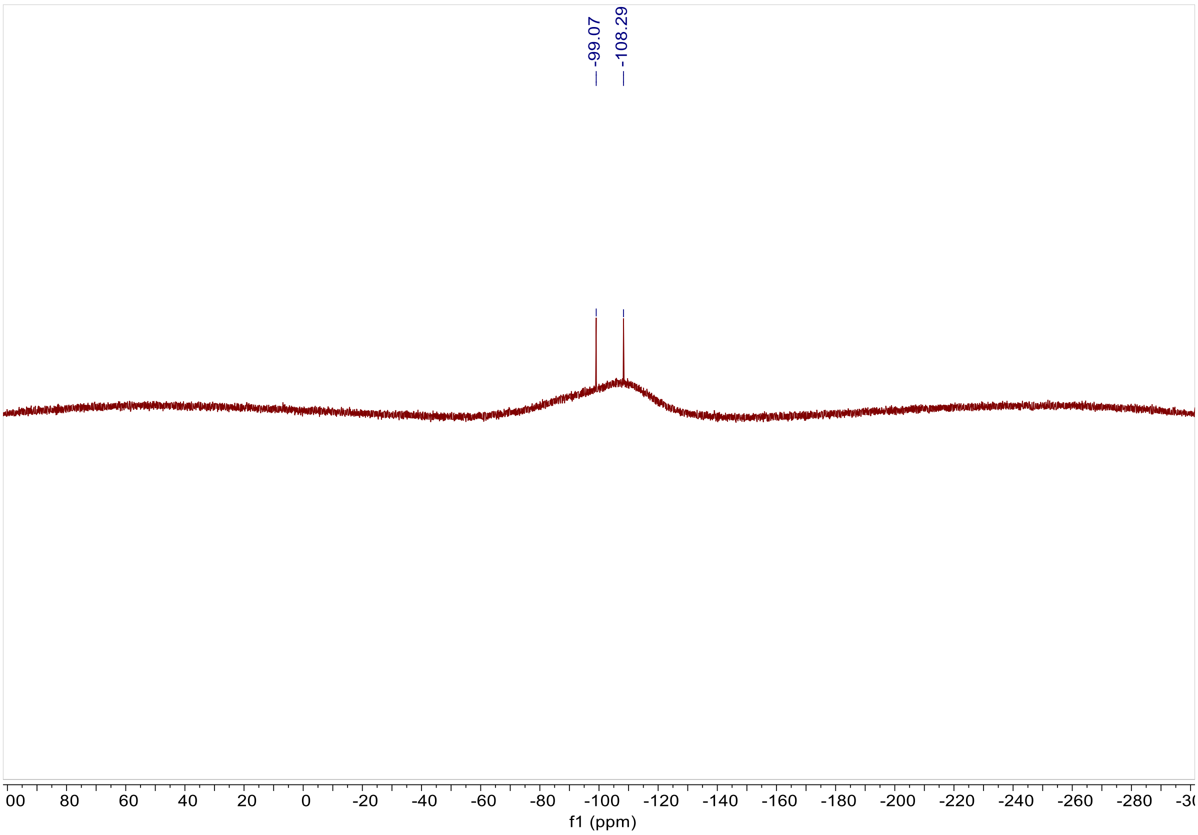

Figure S21. $^{29}Si\{^1H\}$ NMR (79.5 MHz, 298 K) spectrum of **5** in C_6D_6.

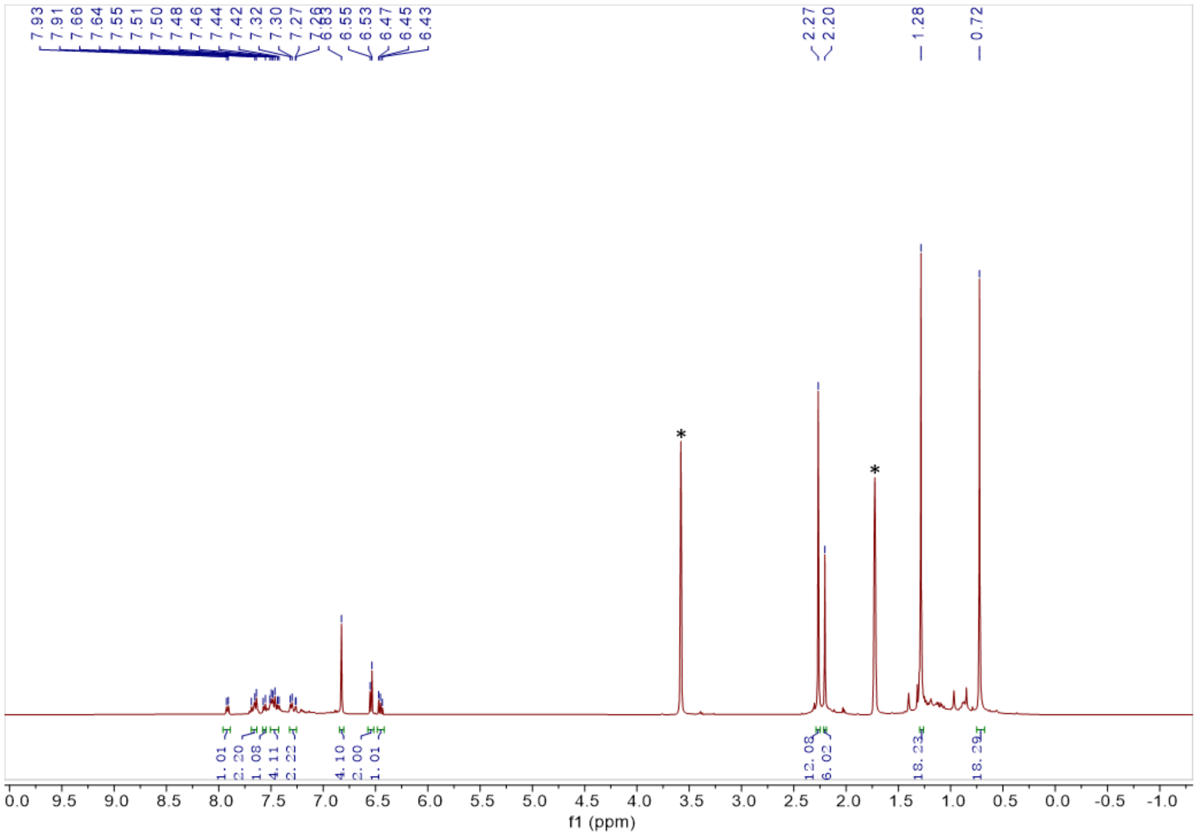

Figure S22. ^1H NMR spectrum (400 MHz, 298 K) of **6** in THF-d_8. *, residual protio solvent signal.

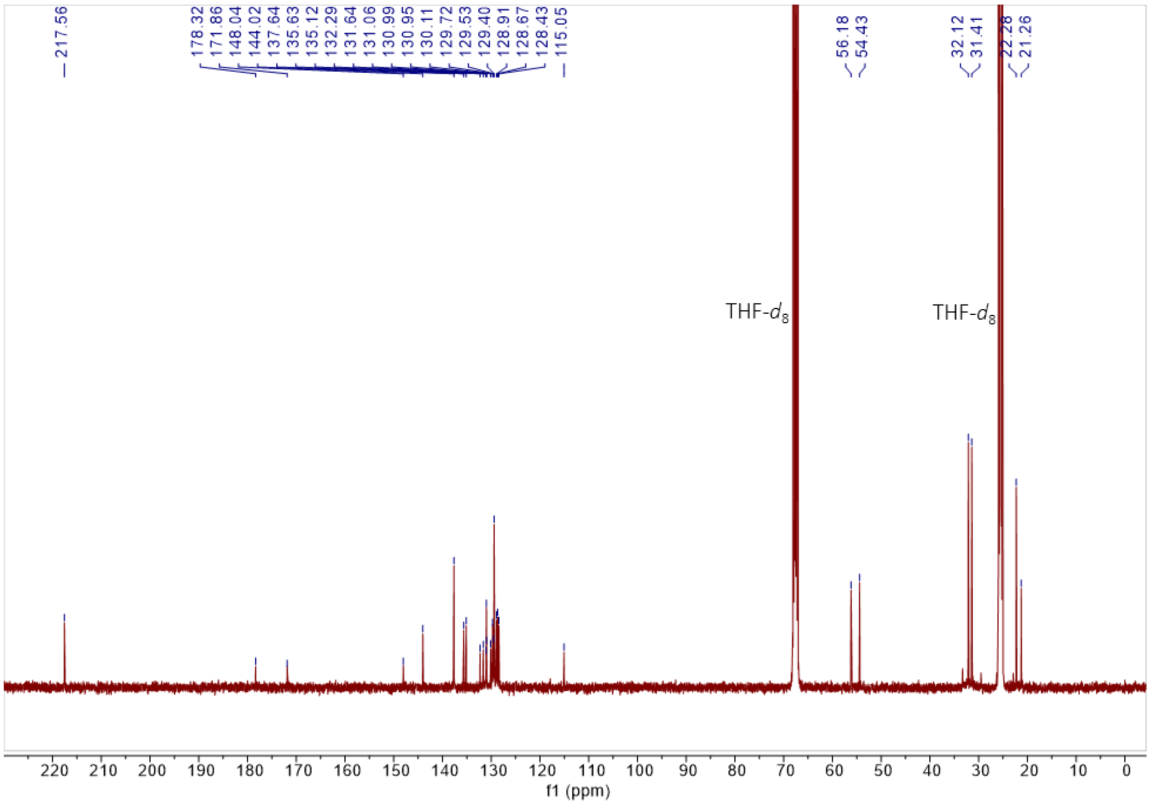

Figure S23. $^{13}C\{^1H\}$ NMR spectrum (101 MHz, 298 K) of **6** in THF-d_8.

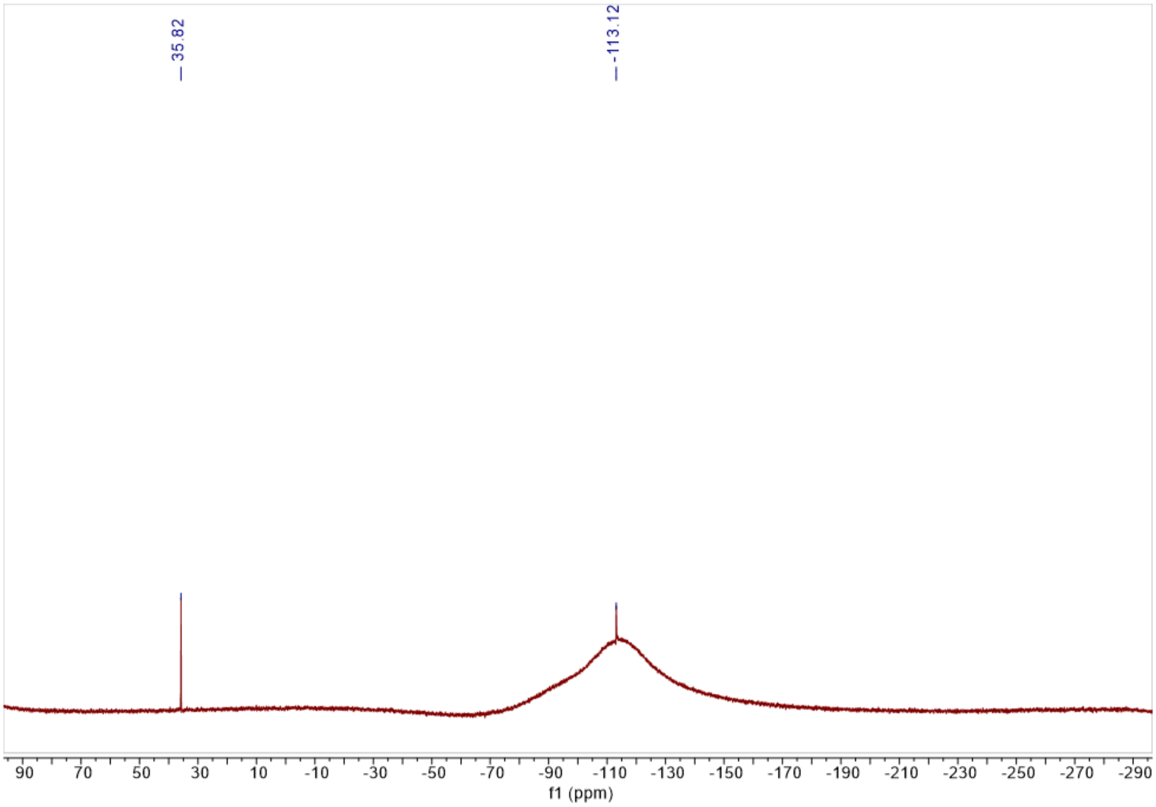

Figure S24. $^{29}Si\{^1H\}$ NMR (79.5 MHz, 298 K) spectrum of **6** in THF-d_8.

3. IR spectra

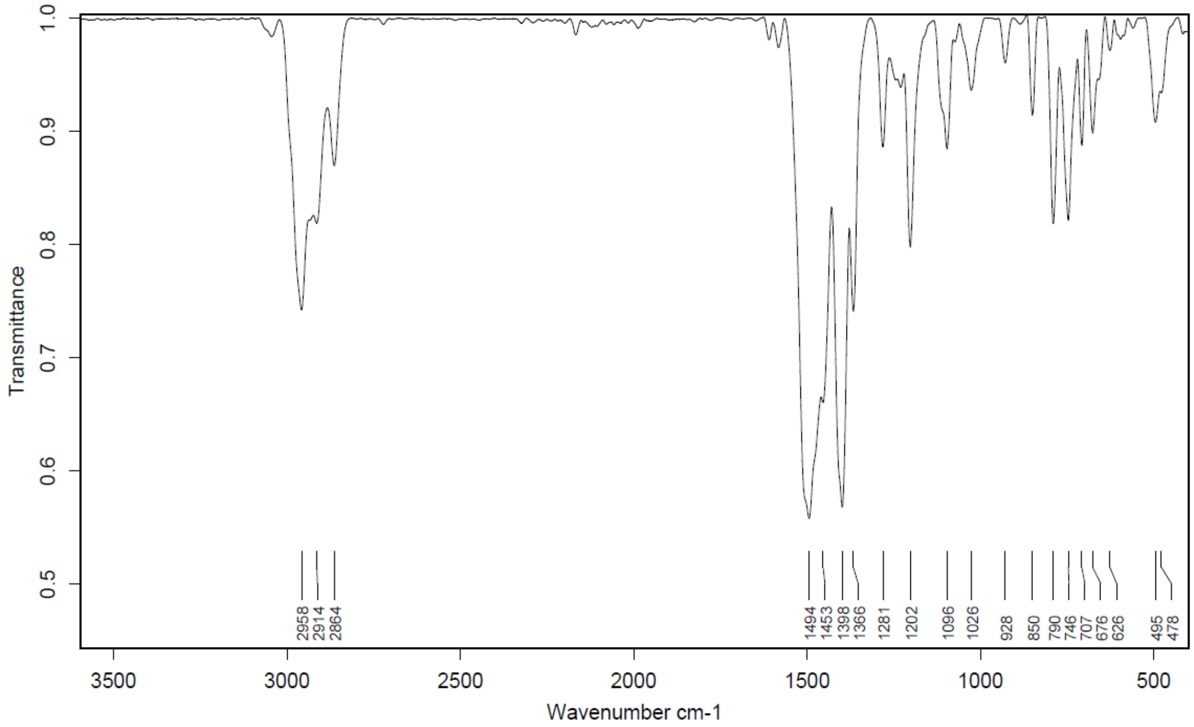

Figure S25. IR spectrum of **1**.

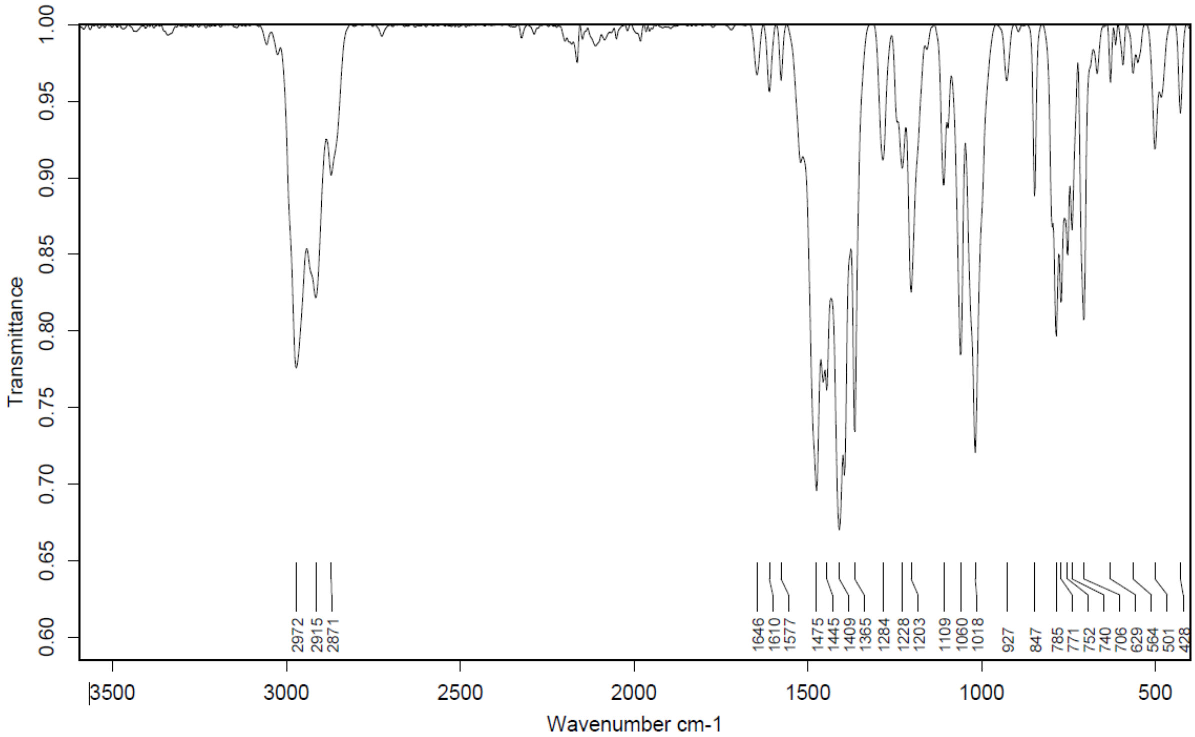

Figure S26. IR spectrum of **3**.

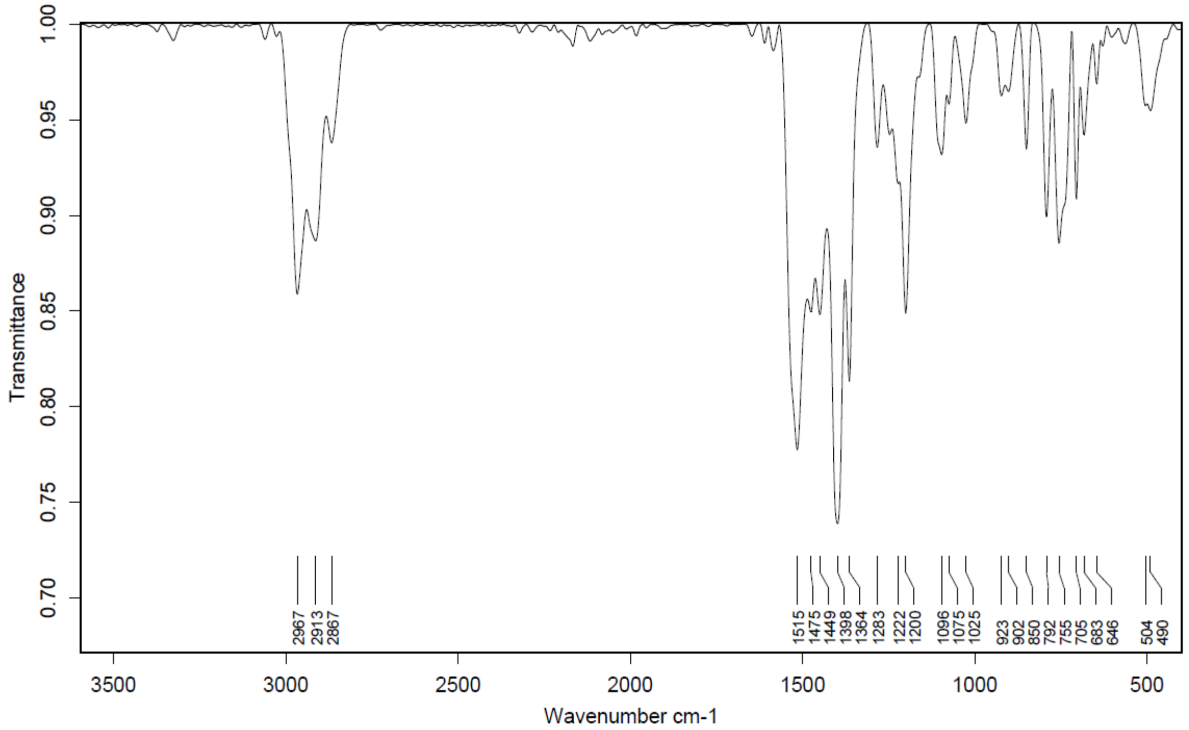

Figure S27. IR spectrum of **5**.

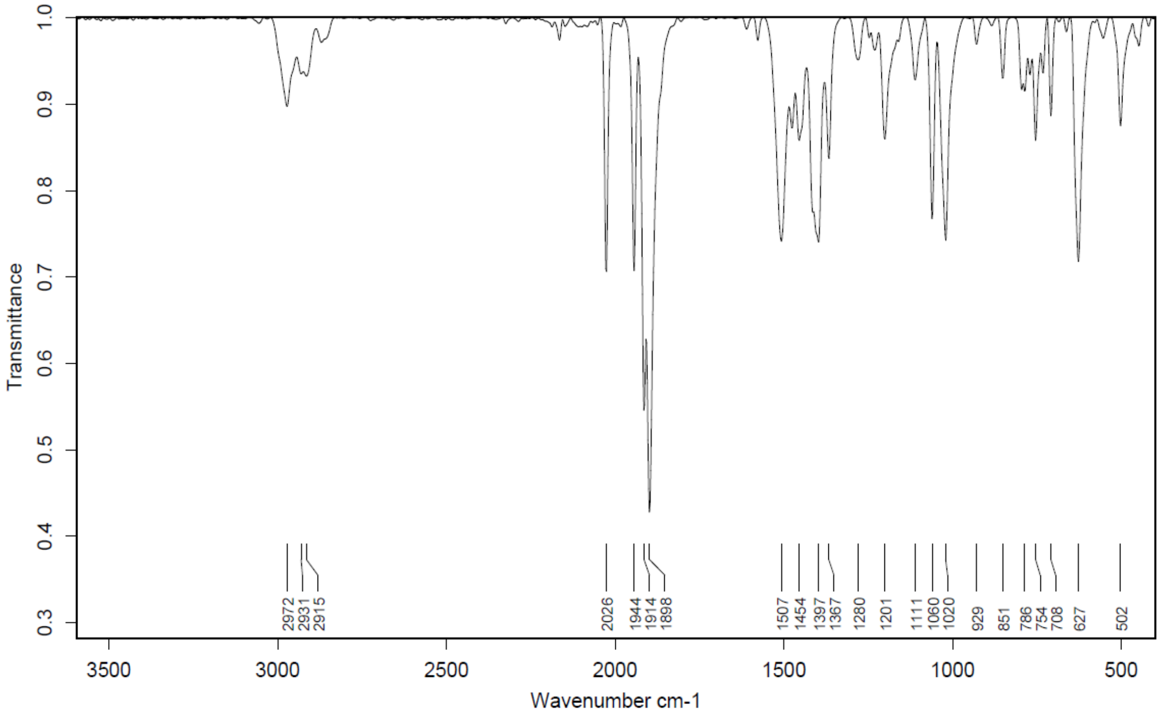

Figure S28. IR spectrum of **6**.

4. X-ray crystallographic studies

4.1 General methods

A suitable crystal was covered in mineral oil (Aldrich) and mounted on a glass fiber. The crystal was transferred directly to the cold stream of a STOE StadiVari diffractometer. All structures were solved by using the program SHELXS/T[8-9] and Olex2.[10] The remaining non-hydrogen atoms were located from successive difference Fourier map calculations. The refinements were carried out by using full-matrix least-squares techniques on F^2 by using the program SHELXL.[8-9] In each case, the locations of the largest peaks in the final difference Fourier map calculations, as well as the magnitude of the residual electron densities, were of no chemical significance. Specific comments for each data set are given below.

Crystallographic data (excluding structure factors) for the structures reported in this paper have been deposited with the Cambridge Crystallographic Data Centre as a supplementary publication no. CCDC 2477857-2477861. Copies of the data can be obtained free of charge on application to CCDC, 12 Union Road, Cambridge CB21EZ, UK (fax: (+(44)1223-336-033; email: deposit@ccdc.cam.ac.uk). Summary of the crystal data, data collection and refinement for compounds are given in Table S1.

The following special comments apply to the models of the structures:

–In the crystal structure of **1**, the *-N=N-* group (N1 and N2), ^{t}Bu group (C57-C60) and Mes group (C43-C51) are disordered over two positions with an occupancy of 0.78/0.22, 0.77/0.23 and 0.53/0.47, respectively.

4.2 Summary of crystal data

Table S1. Crystal data, data collection and refinement for compounds **1**, and **3** to **6**.

Compound	**1**	**3**	**4**	**5**	**6**
Formula	$C_{66}H_{88}N_8Si_2$	$C_{54}H_{71}N_5OSSi_2$	$C_{110}H_{142}N_{10}O_2S_4Si_4 \cdot 2(C_4H_{10}O)$	$C_{62}H_{80}N_8Si_2 \cdot 1.5(C_6H_6)$	$C_{58}H_{71}FeN_5O_5Si_2$
$D_{calc.}$/ g cm^{-3}	1.105	1.033	1.177	1.155	1.247
μ/mm^{-1}	0.101	0.135	0.180	0.103	0.370
Formula Weight	1049.62	894.39	2024.16	1110.68	1030.22
Colour	orange	yellow	yellow	orange	colorless
Shape	plate	block	block	fragment	plate
Size/mm^3	0.03×0.09×0.15	0.47×0.28×0.12	0.29×0.18×0.07	0.31×0.20×0.08	0.27×0.19×0.10
T/K	120	100	100	100	100
Crystal System	monoclinic	monoclinic	monoclinic	triclinic	orthorhombic
Space Group	$P2_1/c$	$P2_1/n$	$P2_1/c$	$P\bar{1}$	$Pbca$
a/Å	11.5273(19)	16.865(3)	19.2707(15)	12.0920(4)	20.7399(7)
b/Å	30.573(3)	14.4507(16)	16.4225(12)	14.4409(4)	21.2084(5)
c/Å	18.591(3)	24.656(5)	36.665(2)	18.4729(5)	24.9535(5)
α/°				94.362(2)	
β/°	105.722(11)	106.845(13)	100.139(5)	92.027(3)	
γ/°				96.335(3)	
V/Å^3	6307.0(15)	5750.9(16)	11422.3(14)	3193.62(16)	10976.0(5)
Z/Z'	4/1	4/1	4/1	2/1	8/1
Wavelength/Å	0.71073	0.71073	0.71073	0.71073	0.71073
Radiation type	MoK_α	MoKa	MoK_α	MoK_α	MoK_α
Θ_{min}/°	1.752	2.226	2.323	1.872	2.306
Θ_{max}/°	29.078	25.250	27.838	30.141	28.315
Measured Refl.	33853	32204	62575	33205	44691
Independent Refl.	14321	10397	25102	15273	12783
Reflections with I > 2(I)	7822	7889	14130	12134	9894
R_{int}/Parameters/Restraints	0.0496/851/442	0.1071/586/0	0.0438/1301/0	0.0282/750/0	0.0390/658/0
Largest Peak/Deepest Hole	0.32/-0.40	0.894/-0.662	0.485/-0.436	0.58/-0.40	0.41/-0.40
GooF	1.023	1.154	1.015	1.024	1.026
wR_2 (all data)	0.1606	0.2931	0.1785	0.1268	0.1274
wR_2	0.1291	0.2643	0.1491	0.1172	0.1179
R_1 (all data)	0.1515	0.1113	0.1214	0.0604	0.0614
R_1	0.0675	0.0945	0.0592	0.0448	0.0443

4.3 Crystal structures

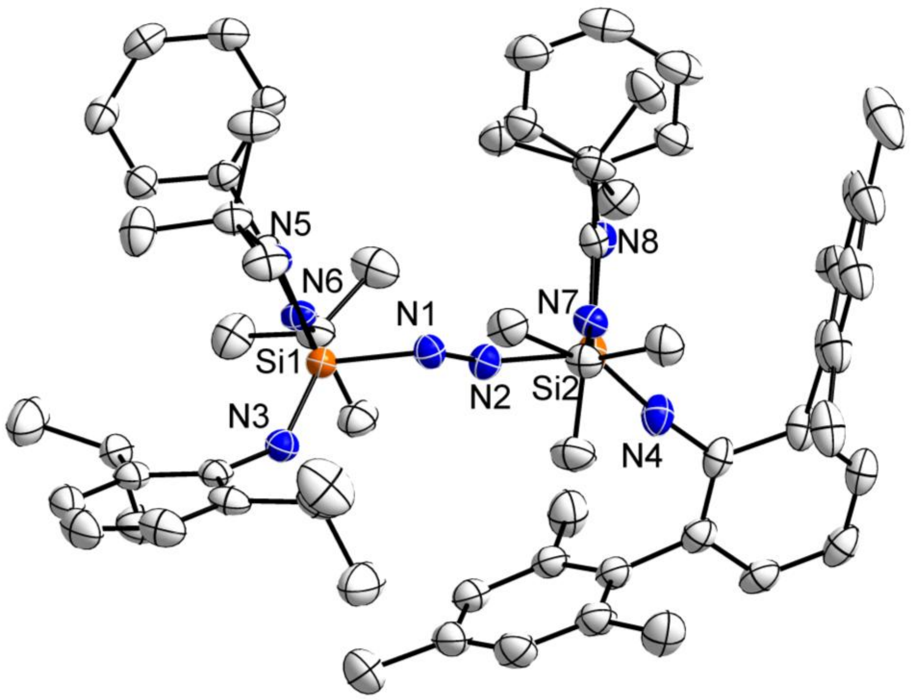

Figure S29. Molecular structure of **1** in the solid state with thermal ellipsoids at the 50% probability level. All hydrogen atoms are omitted for clarity. Selected bond lengths (Å) and bond angles [°]: N1−N2 1.260(2), Si1−N1 1.7939(12), Si1−N3 1.5573(13), Si1−N5 1.8214(12), Si1−N6 1.8211(11), Si2−N2 1.7937(12), Si2−N4 1.5654(12), Si2−N7 1.8208(12), Si2−N8 1.8217(12);

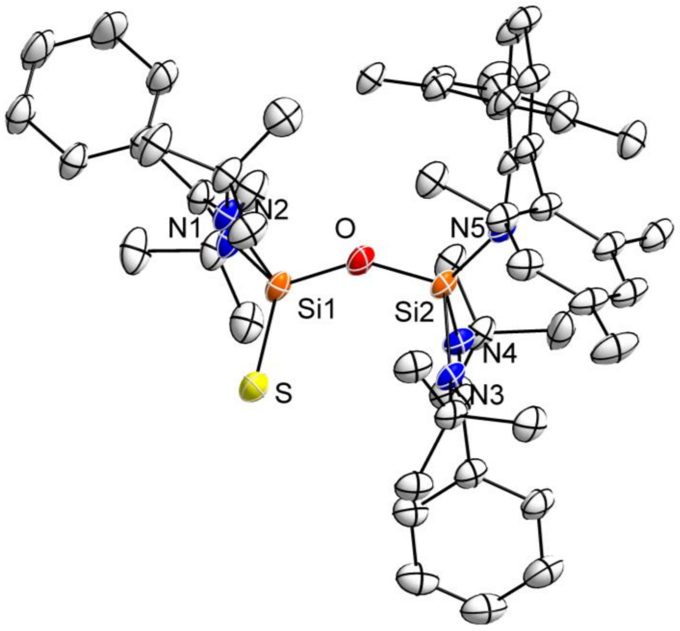

Figure S30. Molecular structure of **3** in the solid state with thermal ellipsoids at the 50% probability level. All hydrogen atoms are omitted for clarity. Selected bond lengths (Å) and bond angles [°]: S−Si1 1.9763(13), Si1−O 1.621(2), Si2−O 1.636(2), Si1−N1 1.826(3), Si1−N2 1.830(3), Si2−N3 1.832(3), Si2−N4 1.815(3), Si2−N5 1.583(3); O−Si1−S 120.74(9), Si1−O−Si2 146.4(2), N1−Si1−N2 71.86(12), N3−Si2−N4 71.79(12).

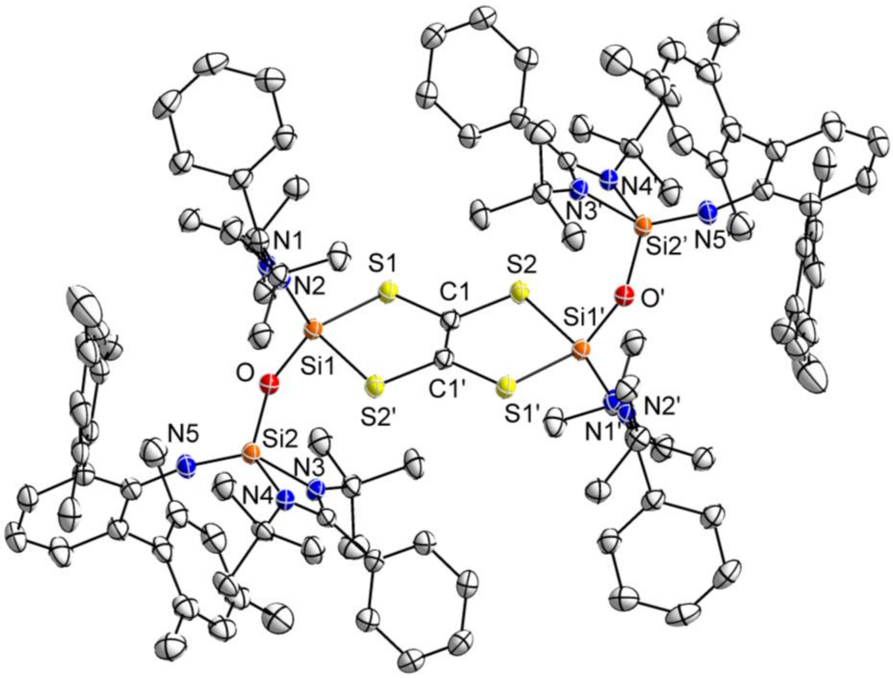

Figure S31. Molecular structure of compound **4** in the solid state with thermal ellipsoids at the 50% probability level. Compound **4** crystallizes with two independent molecules and two Et_2O molecules in the asymmetric unit. As the bond lengths and angles are similar between the two molecules, only one is depicted here. All hydrogen atoms and solvent molecules have been omitted for clarity. Selected bond lengths (Å) and bond angles [°]: S1–Si1 2.1867(9), Si1–S2' 2.2588(11), Si1–O 1.638(2), Si1–N1 1.989(2), Si1–N2 1.822(2), S1–C1 1.759(3), S2–C1 1.752(2), Si2–O 1.636(2), Si2–N3 1.835(2), Si2–N4 1.827(2), Si2–N5 1.569(2), C1–C1' 1.332(5); C1–S1–Si1 104.04(9), S1–Si1–S2' 90.70(4), C1'–S2'–Si1 101.50(10), C1–C1'–S2' 122.4(3), C1'–C1–S1 120.4(3), S1–C1–S2 117.10(15), Si1–O1–Si2 154.67(13).

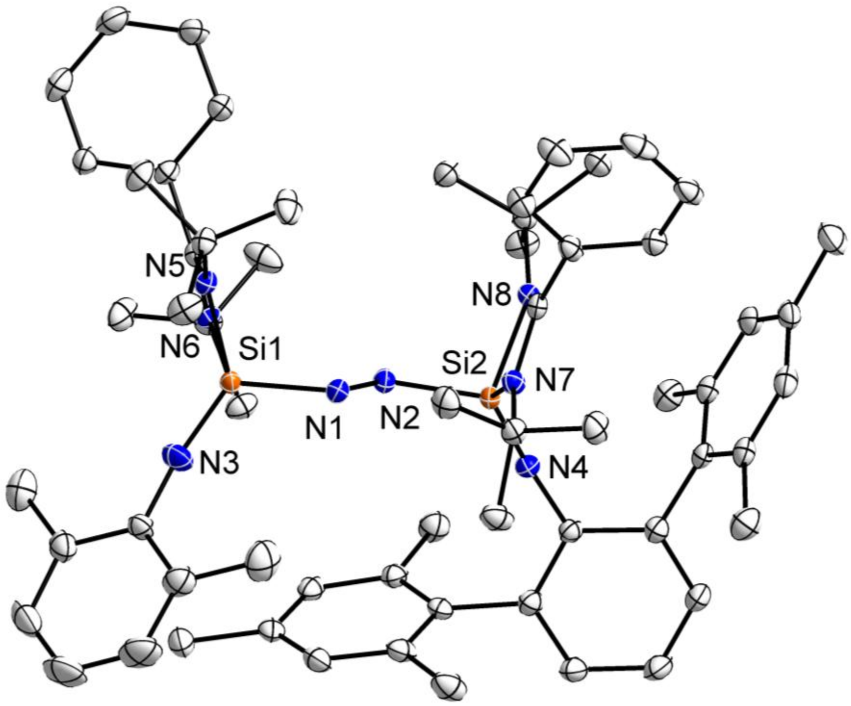

Figure S32. Molecular structure of **5** in the solid state with thermal ellipsoids at the 50% probability level. All hydrogen atoms are omitted for clarity. Compound **5** crystallizes with one and half non-coordinating C_6H_6 molecule in the asymmetric unit. Selected bond lengths (Å) and bond angles [°]: N1−N2 1.260(2), Si1−N1 1.7939(12), Si1−N3 1.5573(13), Si1−N5 1.8214(12), Si1−N6 1.8211(11), Si2−N2 1.7937(12), Si2−N4 1.5654(12), Si2−N7 1.8208(12), Si2−N8 1.8217(12); N1−N2−Si2 116.72(9), N2−N1−Si1 119.27(9), N1−Si1−N3 117.50(7), N5−Si1−N6 72.07(5), N7−Si2−N8 72.24(5), N2−Si2−N4 113.71(6).

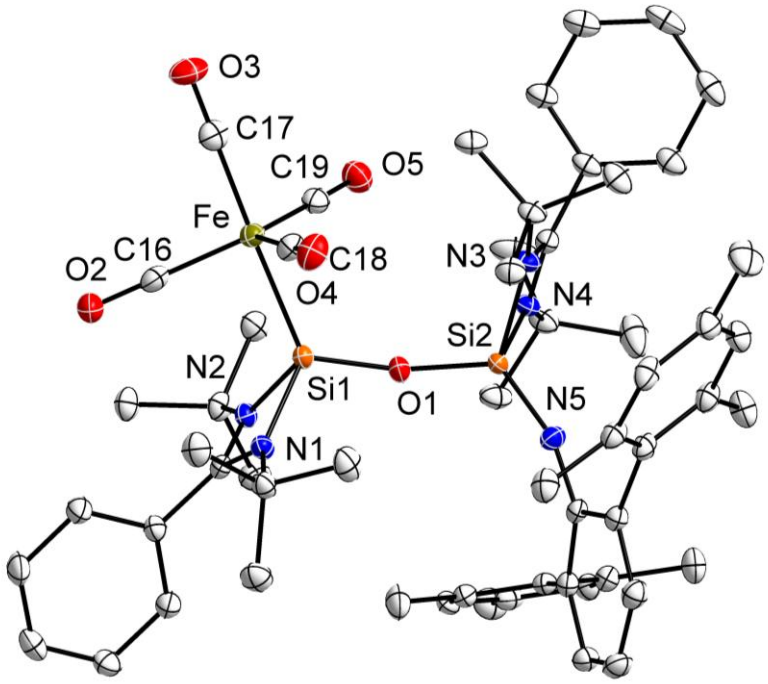

Figure S33. Molecular structure of **6** in the solid state with thermal ellipsoids at the 50% probability level. All hydrogen atoms are omitted for clarity. Selected bond lengths (Å) and bond angles [°]: Fe−Si1 2.2472(6), Fe−C16 1.776(2), Fe−C17 1.789(2), Fe−C18 1.779(2), Fe−C19 1.773(2), O2−C16 1.158(2), O3−C17 1.148(2), O4−C18 1.154(2), O5−C19 1.160(3), Si1−O1 1.6209(13), Si1−N1 1.830(2), Si1−N2 1.8376(15), Si2−O1 1.6308(13), Si2−N3 1.8342(15), Si2−N4 1.822(2), Si2−N5 1.571(2); C16−Fe−Si1 87.00(6), C17−Fe−Si1 174.43(7), C18−Fe−Si1 90.33(6), C19−Fe−Si1 82.81(6), Si1−O1−Si2 166.35(9), O1−Si1−Fe 121.59(5), N1−Si1−N2 71.52(7), O1−Si2−N3 108.19(7), O1−Si2−N4 108.37(7), O1−Si2−N5 121.01(8), N3−Si2−N4 71.83(7).

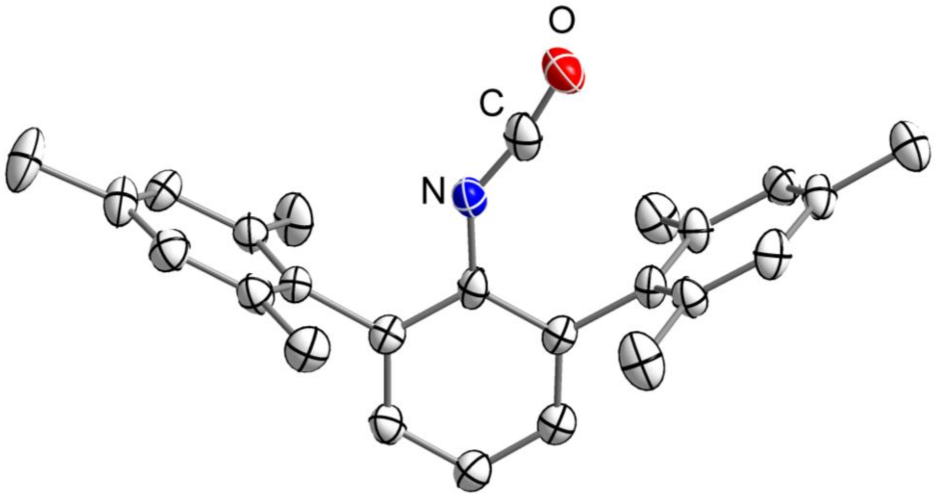

Figure S34. Molecular structure of **TerNCO** in the solid state with thermal ellipsoids at the 50% probability level. All hydrogen atoms are omitted for clarity. Although a single crystal was obtained and measured, a full crystallographic refinement was not performed, as the identity of the compound was already established via NMR and consistent with reported data.

5. Computational details

All computations were performed using Gaussian16[11] utilizing the PBE1PBE level of theory. Structure optimisation was done with Def2-SVP basis sets and empirical dispersion correction (GD3BJ) as implemented in the software suite. The geometry was then used for a single point calculation with Def2-TZVP basis set. No solvent corrections were applied. All optimized molecular structures were checked to be minima on the energy hypersurface and possess no imaginary vibrational frequencies, while transition states feature exactly one negative frequency along the reaction coordinate. For the transition states, IRC calculations were carried out to confirm that they connect the desired intermediates. TS1, TS12, TS16, and TS17 remain unidentified.

Reaction pathway for CO activation

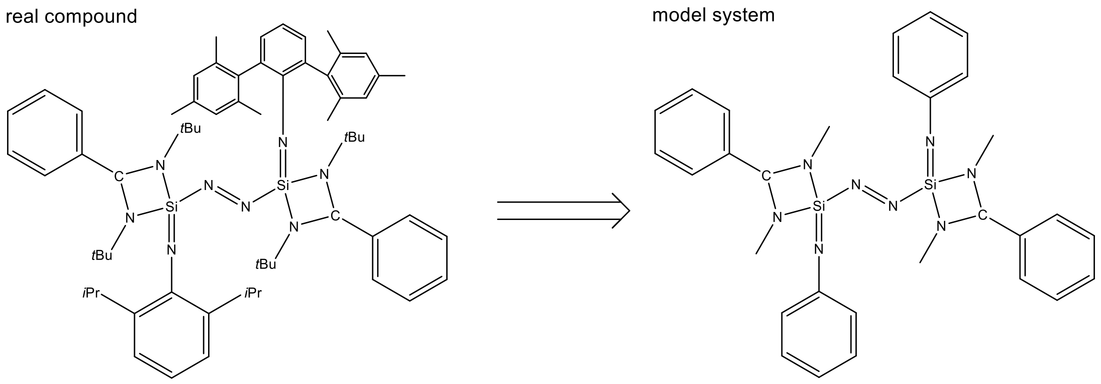

Figure S35. Model system.

For the exploration of the reaction pathway, model compounds with reduced steric bulky were used. Tert-butyl groups were replaced by methyl, Dipp and terphenyl were replaced by phenyl groups. Three discernible sequential phases can be identified in the process: CO addition, N_2 elimination and isonitrile elimination.

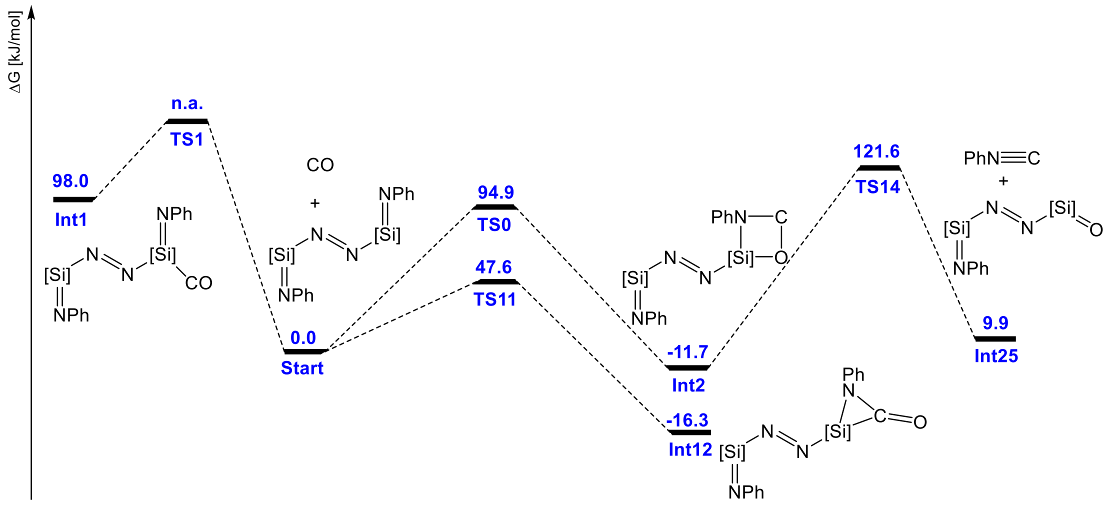

Figure S36. Reaction energy profile for CO addition.

Part 1: CO addition

The first step of the addition of CO is likely to proceed via [2+2] cycloaddition and formation of a NCOSi heterocycle (Start-TS0-Int2, Fig. S36). The immediate alternate cycloreversion with concomitant isonitrile formation is essentially thermoneutral but features a high activation barrier (TS14), therefore this path is unlikely to occur.

Alternatives to [2+2] cycloaddition are [2+1] cycloaddition or coordination of CO at the acidic Si center. These competing pathways are either high in energy as in the case of Int1 (coordination of CO at the acidic Si center) or reversible as for Int12 ([1+2] cycloaddition and formation of NCSi heterocycle). The product of [2+1] cycloaddition, Int12, could be a valid intermediate for further reactions, but no productive pathway could be identified.

Int2 is a plausible intermediate on the productive but more complex reaction pathway and further studied in the reaction sequence.

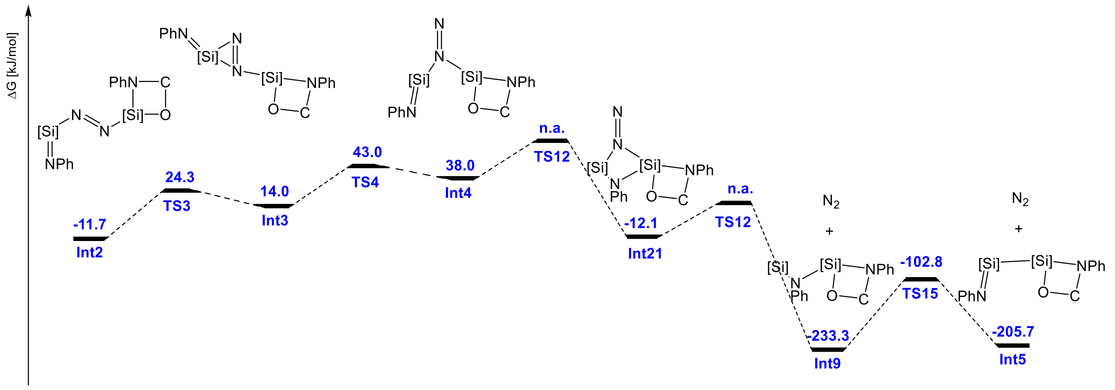

Figure S37. Reaction energy profile for N_2 elimination.

Part2: N_2 elimination

The elimination of N_2 is the result of a stepwise rearrangement. Initially, the non-CO bound silyl group migrates along the N_2 unit which is a slightly endergonic process with low activation barriers (Int2 – Int4). Subsequently, the imino function attacks the other silyl group and eliminates N_2. While the N_2Si_2 cyclic intermediate could be found, the corresponding transition states eluded identification. N_2 elimination results strongly exergonic and the driving force of the reaction. The product Int9 can rearrange to yield the disilane Int5 with a modest activation barrier.

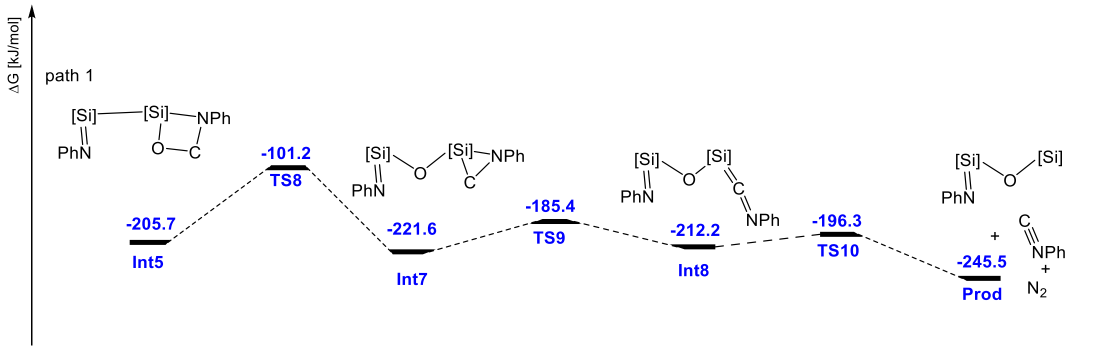

Figure S38. Reaction energy profile for isonitrile elimination (option 1).

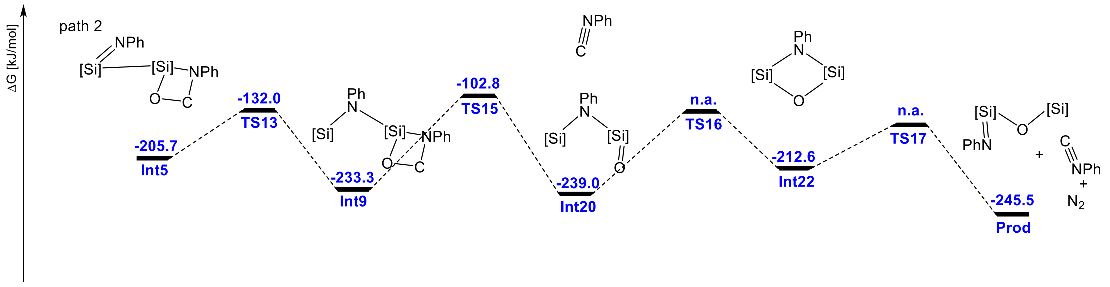

Figure S39. Reaction energy profile for isonitrile elimination (option 2).

Part3: Isonitrile elimination

Isonitrile elimination is the final step in the reaction sequence. Two paths with plausible energy profiles were found.

Option 1: Int5 can undergo silyl attack on the oxygen atom which forms Int7. The side-on bound isonitrile rearranges with low activation barrier to terminal binding mode and elimination, yielding the product.

Option 2: Alternatively, the silyl-silylene Int9 can directly eliminate the isonitrile, yielding the silanone Int20. Ring closure and opening via Int22 yield the product as well, but the transition states for this last process remain unidentified.

6. References

[1] R. Yadav, X. Sun, R. Köppe, M. T. Gamer, F. Weigend, P. W. Roesky, *Angew. Chem. Int. Ed.* **2022**, *61*, e202211115.

[2] J. Bresien, C. Hering-Junghans, A. Schulz, M. Thomas, A. Villinger, *Organometallics* **2018**, *37*, 2571-2580.

[3] M. C. Dietl, V. Vethacke, A. Keshavarzi, F. F. Mulks, F. Rominger, M. Rudolph, I. A. I. Mkhalid, A. S. K. Hashmi, *Organometallics* **2022**, *41*, 802-810.

[4] B. F. Wicker, J. Scott, A. R. Fout, M. Pink, D. J. Mindiola, *Organometallics* **2011**, *30*, 2453-2456.

[5] U. J. Kilgore, F. Basuli, J. C. Huffman, D. J. Mindiola, *Inorg. Chem.* **2006**, *45*, 487-489.

[6] C. A. Laskowski, A. J. M. Miller, G. L. Hillhouse, T. R. Cundari, *J. Am. Chem. Soc.* **2011**, *133*, 771-773.

[7] J. Du, L. Wang, M. Xie, L. Deng, *Angew. Chem. Int. Ed.* **2015**, *54*, 12640-12644.

[8] G. Sheldrick, *Acta Crystallogr. Sect. A* **2008**, *64*, 112-122.

[9] G. Sheldrick, *Acta Crystallogr. Sect. C* **2015**, *71*, 3-8.

[10] O. V. Dolomanov, L. J. Bourhis, R. J. Gildea, J. A. K. Howard, H. Puschmann, *J. Appl. Crystallogr.* **2009**, *42*, 339-341.

[11] M. J. Frisch, G. W. Trucks, H. Schlegel, G. E. Scuseria, M. A. Robb, J. R. Cheeseman, G. Scalmani, V. Barone, G. A. Petersson, H. Nakatsuji. Gaussian 16, Revision C. 01. Gaussian, Inc., Wallingford CT. 2016.
